# miR-374a-5p regulates inflammatory genes and monocyte function in patients with inflammatory bowel disease

**DOI:** 10.1084/jem.20211366

**Published:** 2022-04-01

**Authors:** Carlos Perez-Sanchez, Ariana Barbera Betancourt, Paul A. Lyons, Zinan Zhang, Chenqu Suo, James C. Lee, Eoin F. McKinney, Louise K. Modis, Christian Ellson, Kenneth G.C. Smith

**Affiliations:** 1 Cambridge Institute of Therapeutic Immunology and Infectious Disease, Jeffrey Cheah Biomedical Centre, Cambridge Biomedical Campus, Cambridge, UK; 2 Department of Medicine, University of Cambridge School of Clinical Medicine, Cambridge Biomedical Campus, Cambridge, UK; 3 Rheumatology Service, Reina Sofia University Hospital, Maimonides Biomedical Research Institute of Córdoba, University of Cordoba, Cordoba, Spain; 4 Molecular Development of the Immune System Section, Laboratory of Immune System Biology and Clinical Genomics Program, National Institute of Allergy and Infectious Diseases, National Institutes of Health, Bethesda, MD; 5 Wellcome Sanger Institute, Wellcome Genome Campus, Cambridge, UK; 6 Department of Paediatrics, Cambridge University Hospitals, Cambridge, UK; 7 Adaptive Immunity Research Unit, GSK, Stevenage, UK; 8 Department of Medicine, Yong Loo Lin School of Medicine, National University of Singapore, Singapore

## Abstract

MicroRNAs are critical regulators of gene expression controlling cellular processes including inflammation. We explored their role in the pathogenesis of inflammatory bowel disease (IBD) and identified reduced expression of miR-374a-5p in IBD monocytes that correlated with a module of up-regulated genes related to the inflammatory response. Key proinflammatory module genes, including for example *TNFα*, *IL1A*, *IL6*, and *OSM*, were inversely correlated with miR-374a-5p and were validated in vitro. In colonic biopsies, miR-374a-5p was again reduced in expression and inversely correlated with the same inflammatory module, and its levels predicted subsequent response to anti-TNF therapy. Increased miR-374a-5p expression was shown to control macrophage-driven inflammation by suppressing proinflammatory mediators and to reduce the capacity of monocytes to migrate and activate T cells. Our findings suggest that miR-374a-5p reduction is a central driver of inflammation in IBD, and its therapeutic supplementation could reduce monocyte-driven inflammation in IBD or other immune-mediated diseases.

## Teaser

miR-374a-5p is a central regulator in IBD, as its down-regulation increases the expression of a module of key inflammatory mediators. miR-374a-5p up-regulation in vitro ameliorated the increased monocyte-driven inflammation by reducing proinflammatory mediators, migration capacity, and T cell activation. miR-374a-5p has a therapeutic potential in immune-mediated diseases.

## Introduction

Inflammatory bowel disease (IBD) is a common, incurable inflammatory disease, comprising Crohn’s disease (CD) and ulcerative colitis (UC). Worldwide, >6.5 million people have IBD, which has its peak onset in young adulthood. Chronic disabling symptoms often impact quality of life and create a substantial economic burden (Alatab et al., 2020), and so understanding pathogenesis to drive new treatment strategies is a priority.

Macrophages and monocytes play central roles in IBD pathogenesis (Na et al., 2019; Baillie et al., 2017) through the production of inflammatory cytokines such as *IL1β*, *IL6*, *IL23*, and *TNFα*. These cytokines recruit and activate other immune cells and are the targets of effective current therapies (Podolsky, 2002; Neurath, 2019). Nonetheless, the molecular mechanisms underlying macrophage and monocyte dysregulation in IBD remain unclear. The analysis of circulating monocytes and derived macrophages from patients is difficult, as their gene expression and function are exquisitely sensitive to immunosuppressive therapies (Lyons et al., 2010). To avoid these confounding effects of treatment, it is necessary to study newly diagnosed, treatment-naive patients.

High-throughput technologies have provided important insights into disease—for example, disease-related transcriptional changes have been identified in mucosal biopsies of IBD patients (Häsler et al., 2017; Hong et al., 2017)—however, the cellular heterogeneity of biopsy samples can hamper interpretation. The analysis of purified cell types can provide a clearer vision of the molecular pathways associated with the disease; for example, transcriptomic analysis of purified CD8^+^ T cells from treatment-naive patients with active IBD revealed signatures that predicted clinical outcome and have led to the development of clinically validated biomarkers (Biasci et al., 2019).

From a therapeutic perspective, the challenge is to make biological sense of large-scale data to identify key upstream regulators. Network analysis of mRNA data identifies modules of coexpressed genes that may correlate with disease features. The identification of mechanisms orchestrating this coregulation could reveal important drivers of immune dysregulation in disease, which in turn might provide potential therapeutic targets. microRNAs (miRs) are attractive candidates, since they are an important class of regulatory molecules that can have widespread effects on multiple RNA transcripts in immune and other contexts (Baltimore et al., 2008). In this study, we sought to identify miRs important in regulating the inflammatory response in monocytes in IBD.

The contribution of miRs to the pathogenesis of IBD is incompletely understood. Most studies have restricted their focus to miRs targeting genes that had been previously linked to the disease at the genetic level (Schaefer, 2016; Kalla et al., 2015). Others have found miR profiles differentially expressed (DE) in intestinal biopsies, blood, or serum (Kalla et al., 2015). Many of those, including miR-21, -146, and -155, are dysregulated in other immune-mediated diseases, but their roles in pathogenesis are yet to be fully elucidated (Kalla et al., 2015).

Discovery of the role played by miRs in driving IBD-associated transcriptional modules will require integrated analysis of miR and mRNA derived from well characterized patients with active disease. To avoid the confounding effects of therapy, only newly diagnosed, treatment-naive patients were included in this study. We performed such an analysis on mRNA and miR quantified from purified blood monocytes and other immune cell types. This analysis found miR-374a-5p to be reduced in blood and colonic biopsies in active IBD, leading to increased expression of key proinflammatory target genes. miR-374a-5p up-regulation in vitro ameliorated the increased monocyte-driven inflammation by reducing proinflammatory mediators, migration capacity, and T cell activation. miR-374a-5p may have a potential therapeutic role in IBD and other immune-mediated diseases.

## Results

### Monocyte gene modules associated with IBD

**Figure 1.**
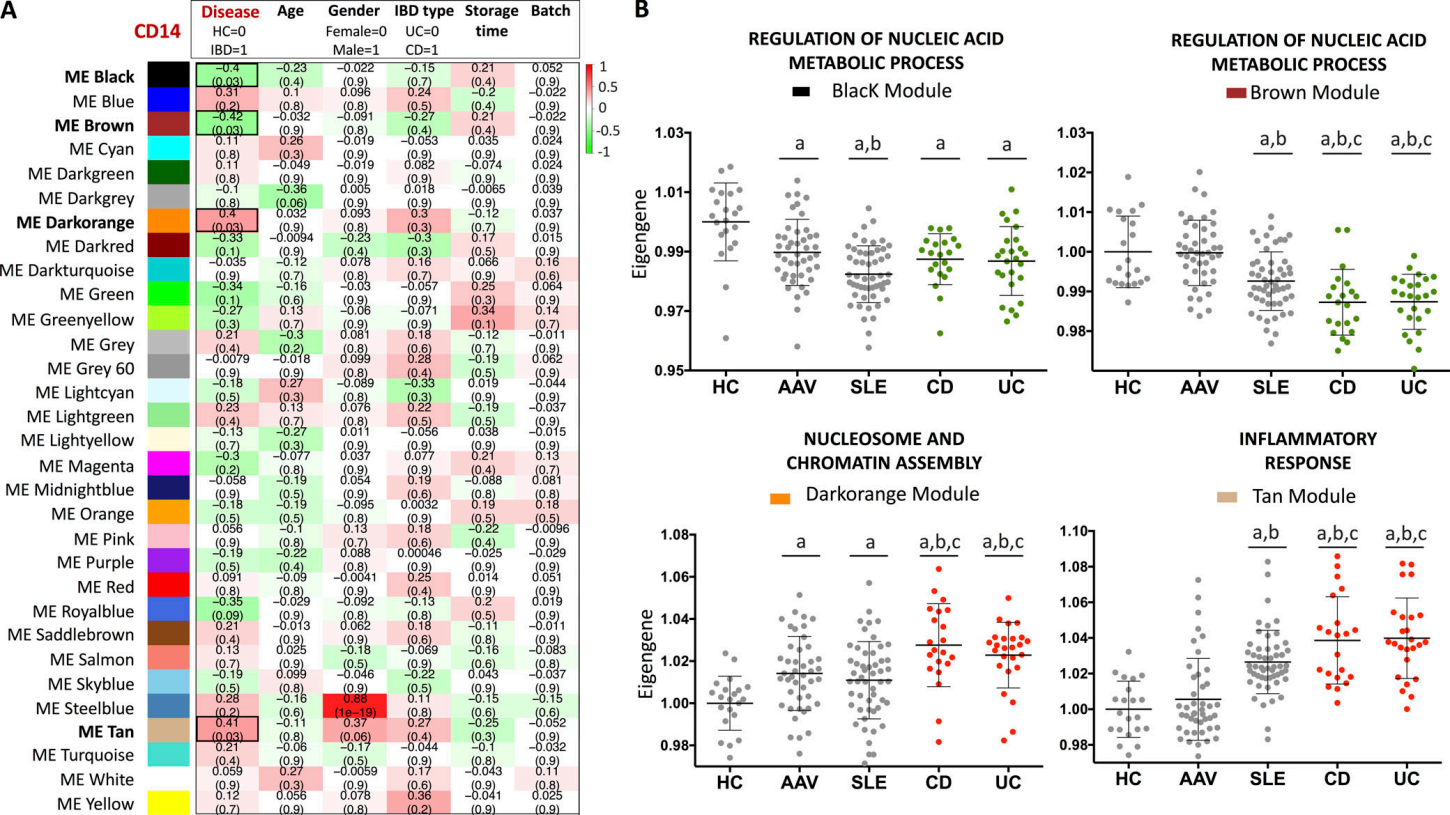
**WGCNA of CD14 monocyte transcriptomes from IBD patients and HC identified RNA modules associated with disease. (A)** The eigengene of each module (ME) was correlated to disease classification and other clinical and analytical variables. The correlation coefficient and significance (FDR) are indicated for each module. Red and green colors represent positive and negative correlations, respectively. **(B)** Eigengene values of the modules across HC and immune-mediated diseases. The eigengene values were normalized to HC. ^a^, P < 0.05 vs. HC; ^b^, P < 0.05 vs. AAV; ^c^, P < 0.05 vs. SLE. One-way ANOVA and Tukey’s post hoc test.

**Figure S1.**
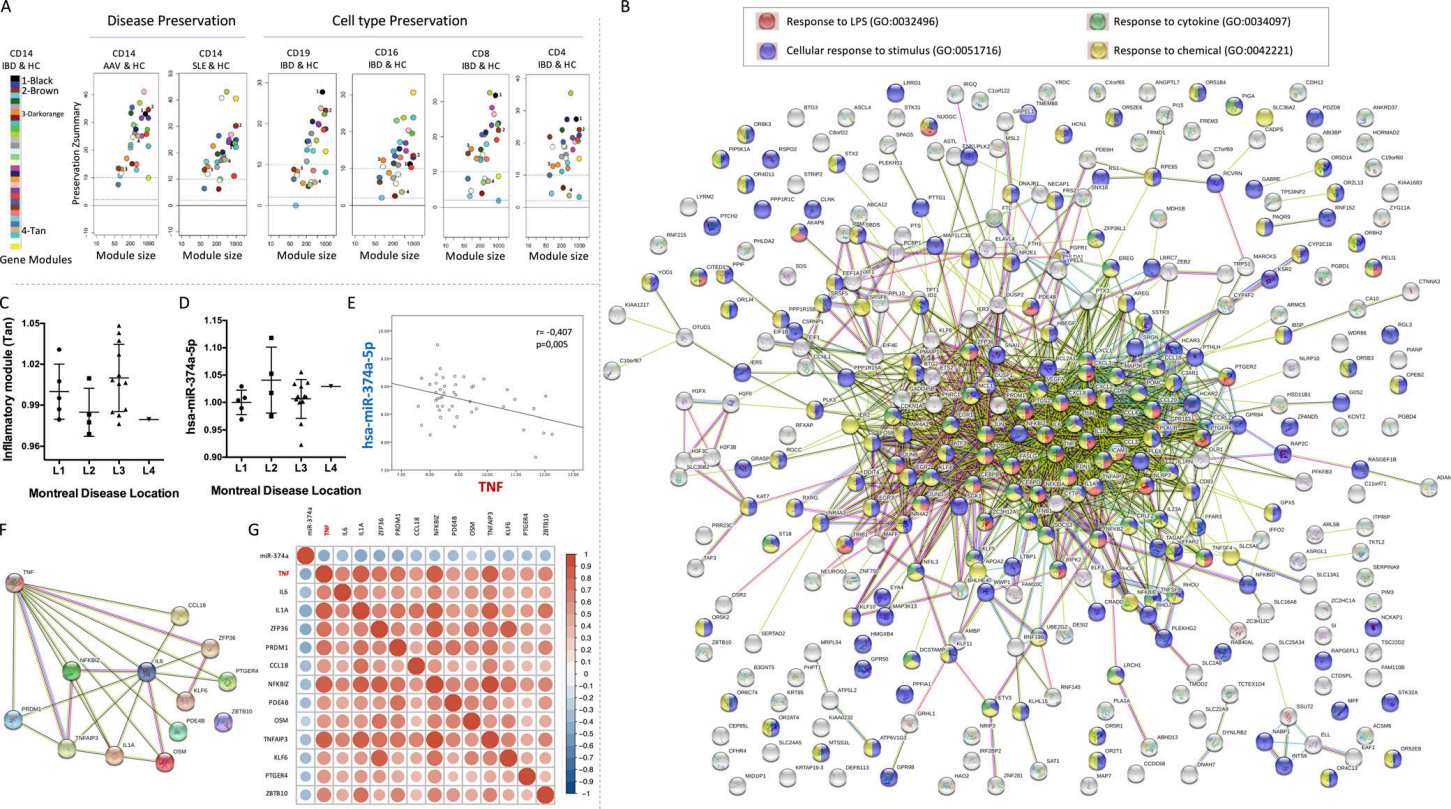
**Module preservation analysis, inflammatory Tan module description, and TNFα associations. (A)** Module preservation of the CD14^+^ IBD-HC network across multiple datasets. The CD14^+^ IBD-HC network was used as the reference, whereas the test datasets included transcriptomes derived from other related diseases (AAV and SLE) and other cell types (CD19^+^, CD16^+^, CD8^+^, and CD4^+^). The Zsummary preservation statistics (y axis) of the modules were used to assess the overall significance of the preservation analysis. Each dot represents a module labeled by color. The dashed blue and green lines indicate the thresholds. A Zsummary value <2 indicates no preservation; a value >2 represents moderate preservation; and a value >10 indicates strong evidence of preservation. **(B)** Tan module associated with inflammatory response. Gene Ontology enrichment analysis and network of known and predicted protein–protein interaction by String platform analysis. Red dots, genes related to the response to LPS; purple dots, genes related to cellular response to stimulus; green dots, genes involved in the response to cytokine; and yellow dots, genes associated with the response to chemical. **(C and D)** Analysis of expression of the Tan module (C) and hsa-miR-374a-5p (D) in monocytes of CD patients assigned by the Montreal disease location classification: L1, terminal ileum; L2, colon; L3, ileocolon; L4, upper gastrointestinal tract. **(E)** Significant inverse correlation between the expression level of miR-374a-5p and TNFα in IBD monocytes. The correlation coefficient and significance are represented. **(F)** Network of known and predicted protein–protein interactions between TNFα and the predicted and inversely correlated targets by using String platform. **(G)** Heatmap of correlations between TNFα, miR-374a-5p, and all the predicted and inversely correlated targets within the inflammatory response module. Red and blue colors represent positive and negative correlations, respectively. The size of the dot corresponds with the correlation coefficient. All correlations included in the heatmap showed P < 0.05. Pearson’s correlation test.

To better understand the contribution of monocytes to IBD pathogenesis, we measured gene expression in purified CD14^+^ monocytes isolated from the peripheral blood of 46 active, untreated IBD patients and 20 age- and sex-matched healthy controls (HC; Table S1). We applied a systems biology method (weighted gene coexpression network analysis [WGCNA]) to help resolve biological networks. Genes were combined into modules based on the pairwise correlation between their expression levels across all samples. 31 discrete modules containing highly coexpressed genes were identified (Fig. 1 A), and module preservation analysis confirmed their existence in other inflammatory diseases (ANCA-associated vasculitis [AAV] and systemic lupus erythematosus [SLE]) and different cell types (B cells, neutrophils, and CD4^+^ and CD8^+^ T cells; Fig. S1 A). We then sought to identify which of these modules were significantly correlated with IBD by excluding those driven by associations with age, sex, sample storage time, etc. This process identified four modules that were significantly associated with IBD, with two containing genes that were up-regulated in disease and two containing genes that were down-regulated. Comparison with other immune-mediated diseases revealed that some, but not all, of these gene sets were similarly dysregulated in other diseases, including SLE and AAV. Significant differences were not observed between CD and UC for any module (Fig. 1, A and B). To understand the biological processes responsible for these transcriptional changes, we used Gene Ontology enrichment analysis and identified that the Black and Brown modules were enriched for genes related to the regulation of nucleic acid metabolic processes, the Darkorange module for genes involved in nucleosome and chromatin assembly, and the Tan module for inflammatory pathways (Figs. 1 B and S1 B). No correlation was found between the Tan module and anatomic location of the disease in CD (Fig. S1 C).

### miR profile of CD14^+^ monocytes from IBD patients

**Figure 2.**
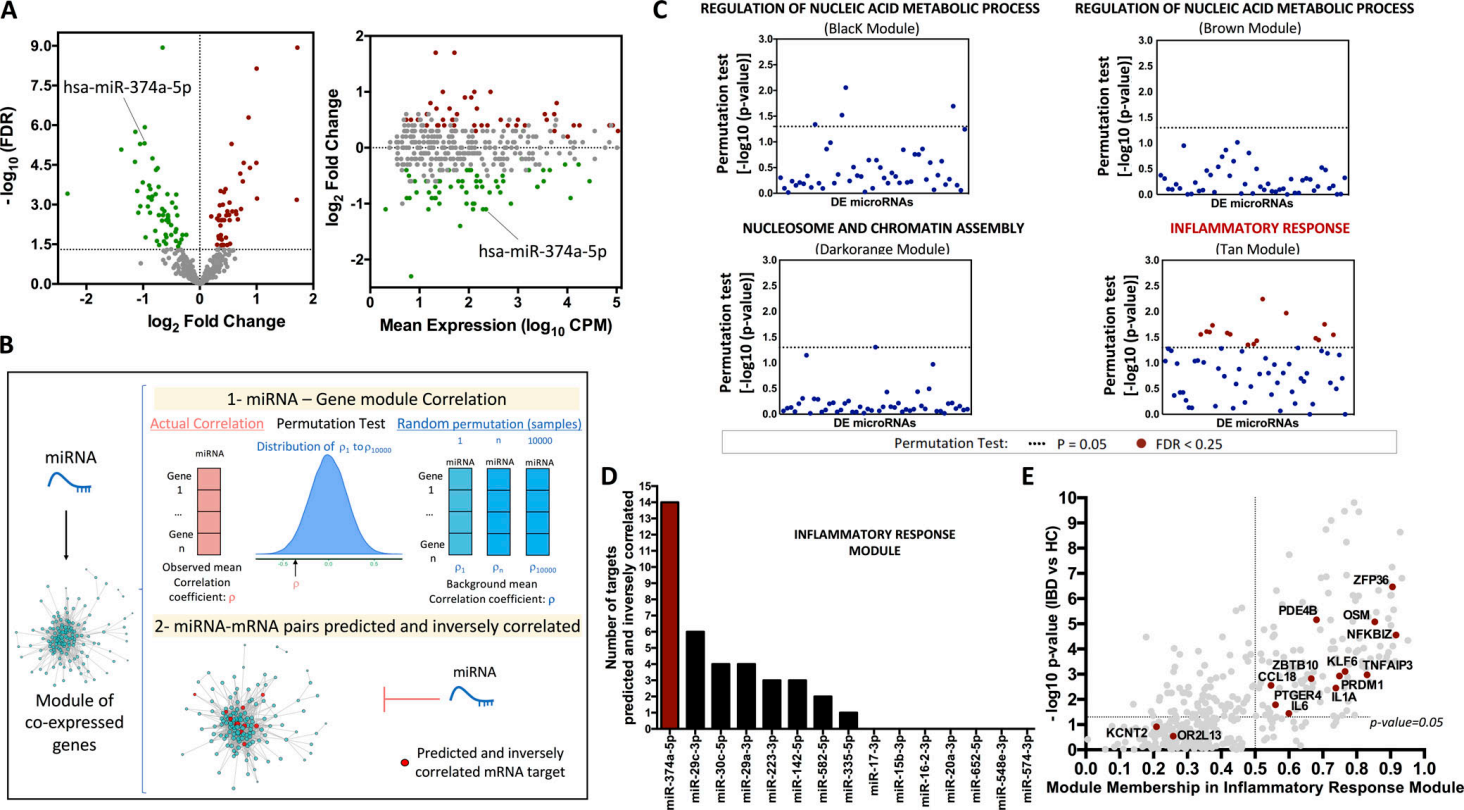
**Integrated mRNA-miR analysis identified miR-374a-5p as a potential key regulator of the inflammatory response in IBD patients. (A)** Volcano plot of miRs DE between IBD and HC in CD14^+^ monocytes (left). Green and red dots represent DE miRs down-regulated and up-regulated in IBD, respectively. Horizontal dashed line indicates P = 0.05. Mean difference plot indicating the mean expression level of miRNAs and their fold-change (right). **(B)** Representative chart summarizing the pipeline of the integrated miRNA-mRNA analysis. The analysis includes two steps: (1) identification of DE miRs that negatively correlate with the genes within the IBD modules by permutation test analysis; and (2) identification of potential targets within the module that are predicted and inversely correlated with those DE miRs. **(C)** First step: permutation test of DE miRs and the IBD modules. Red dots represent DE miRs correlated with the modules considering P < 0.05 and FDR < 0.05. **(D)** Second step: number of predicted and inversely correlated targets within the inflammatory response module for the previously identified miRs. **(E)** Scatter plot showing the relationship between the module membership (MM) of each gene within the inflammatory response module and the differential expression of those genes in IBD compared with HC. Red dots indicate miR-374a-5p potential targets. Genes important in module structure were identified by taking the intersection between differential expression (P < 0.05) and high intramodular connectivity (MM > 0.5).

To determine whether miRs might be contributing to the IBD-associated changes in gene expression, we performed small RNA-sequencing (RNA-seq) analysis in the same samples, to provide a means of comparing miR abundance with changes in overall gene expression. We identified 110 monocyte DE miRs in IBD patients compared with HC (false discovery rate [FDR] ≤ 0.05); 61 miRs were down-regulated and 49 up-regulated (Fig. 2 A). All miRs detected in monocytes are listed in Table S2, and the most significantly DE are highlighted.

### Integrated IBD monocyte mRNA-miR analysis associates miR-374a-5p with the “inflammatory response” module

To further explore the potential impact of miRs on the dysregulation of IBD-associated gene networks, we developed an analysis to integrate the miR and mRNA datasets. This could provide insight into the potential role of miRs in IBD and identify upstream regulators of dysregulated gene sets that might inform therapeutic opportunities (Fig. 2 B). Because miRs usually reduce gene expression (Baltimore et al., 2008), we used a permutation test to select DE miRs that have a significant negative correlation with the genes of a given IBD gene module. 15 down-regulated miRs were correlated with enhanced expression of the inflammatory response (Tan) module, and no significant negative correlations between DE miRs and other modules were found (Fig. 2 C).

We next investigated the enrichment of predicted mRNA targets for each of these 15 miRs in the inflammatory response module. miR-374a-5p, the miR most associated with the module, has more than twice as many predicted and inverse correlated mRNA targets as any other miR (Fig. 2 D). miR-374-5p appears to have a protective role in different pathological conditions, including several cancers (Wu et al., 2016; Chen et al., 2018; Chen et al., 2016), neonatal hypoxic-ischemic encephalopathy (Chen et al., 2020), diabetic nephropathy (Yang et al., 2018), and obesity (Doumatey et al., 2018), among others, and a role for it in controlling inflammatory mediators has been proposed in some of these contexts (Doumatey et al., 2018; Chen et al., 2020). This work adds to these reports in that it suggests miR-374a-5p deficiency could play a previously unrecognized central role in susceptibility to overt immune-mediated diseases such as IBD.

**Figure 3.**
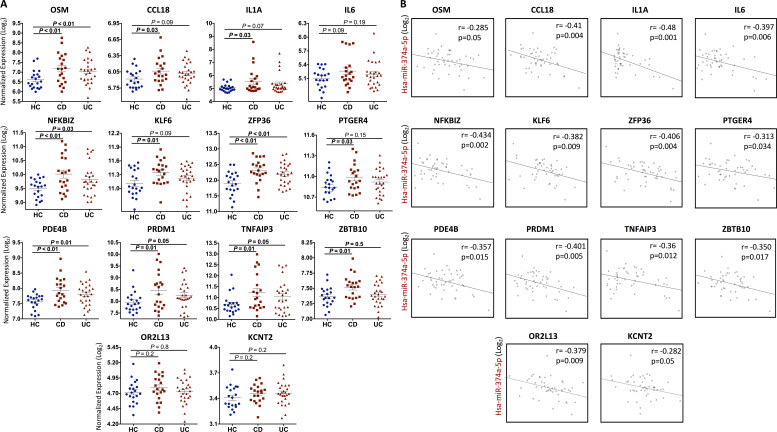
**Differential expression and correlation analysis of miR-374a-5p targets in monocytes from IBD patients. (A)** Expression level of all the predicted and inverse-correlated miR-374a-5p targets within the inflammatory module in monocytes from HC and patients with CD and UC. Mean, SEM, and P are shown. One-way ANOVA and Tukey’s post hoc test. **(B)** Significant inverse correlations between the expression of miR-374a-5p and all the potential predicted targets within the inflammatory response module (Tan module) in monocytes from IBD patients. Pearson’s correlation coefficient and significance are represented in each plot.

Predicted targets of miR-374a-5p include cytokines (*IL1A*, *IL6*, oncostatin M [*OSM*], and *CCL18*), inflammatory transcription factors (*KLF6*, *NFKBIZ*, *PRDM1*, and *ZBTB10*), immune-inflammatory regulators (*TNFAIP3*, *PDE4B*, *PTGER4*, and *ZFP36*), and two genes with no recognized immune-inflammatory functions (*ORL213* and *KCNT2*). Most of these predicted targets are known to play prominent roles in the pathogenesis of IBD (Table S3), underlining the potential importance of regulation of miR-374a-5p. Examples include *OSM*, which has been associated with both disease and treatment response and is a potential therapeutic target (West et al., 2017), and *TNFAIP3* (Bourges et al., 2020). Furthermore, the combined analysis of module membership (MM; the degree to which each gene is associated with the module) and the association of specific gene expression with IBD (defined as minus log P value between IBD and HC by *t* test) revealed that most of these targets were central to module structure, except for *KCNT2* and *OR2L13*, the only two without known roles in inflammation (Fig. 2 E). In addition, alone among the potential targets of miR-374a-5p, only *KCTN2* and *OR2L13* showed no evidence of differential expression between IBD and health, and so they will not be considered further. No differences in target gene expression were seen between CD and UC (Fig. 3 A). The inverse correlation between miR-374a-5p and its predicted targets is shown in Fig. 3 B. No correlation was found between miR-374a-5p and anatomic location of the disease in CD (Fig. S1 D).

*TNFα*, a key cytokine involved in the pathogenesis of IBD, is within the inflammatory response module. It is likely to be a coregulated gene, as while its expression correlated inversely with miR-374a-5p, it lacked a predicted seed sequence motif (Fig. S1, E–G; and see Discussion).

Other miRs have been previously associated with IBD, including miR-31 (Keith et al., 2018), miR-200c (Rawat et al., 2020), and miR-142 (Schaefer et al., 2015). We found that miR-142-5p and miR-142-3p are highly expressed in monocytes and significantly down-regulated in IBD compared with HC (Table S2), whereas miR-31-5p and miR-200c-3p were lowly expressed in monocytes and not DE in IBD. As these miRs were not associated with gene expression modules, they are not considered further.

### miR-374a-5p and the inflammatory response module in different cell types

**Figure 4.**
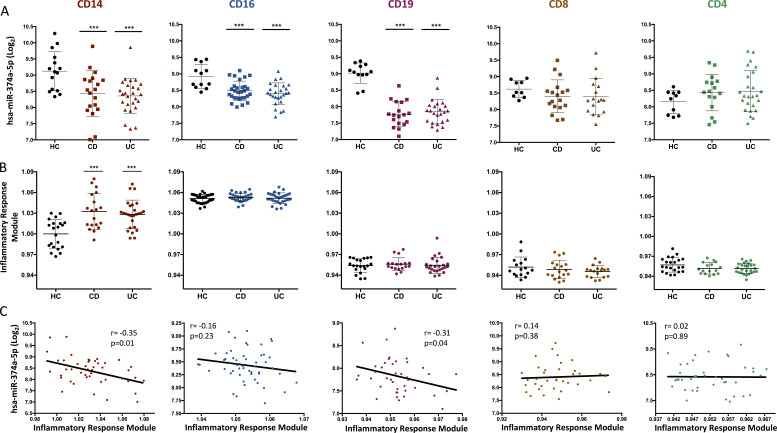
**Relationship between miR-374a-5p and the inflammatory response module in different cell types from HC and IBD patients. (A)** Expression level of miR-374a-5p between HC, CD, and UC across five different cell types. Mean and SD are represented. **(B)** Eigengene level of the inflammatory response module between HC and patients with CD and UC across five different cell types (CD14^+^, CD16^+^, CD19^+^, CD8^+^, and CD4^+^). Eigengene values were normalized to the CD14^+^ HC group. ***, P < 0.001. One-way ANOVA and Tukey’s post hoc test. **(C)** Correlation between the level of miR-374a-5p and the eigengene of the inflammatory response module in IBD patients. Pearson’s correlation test.

We next explored the cell type specificity of the association between miR-374a-5p and the inflammatory response module. In HC, miR-374a-5p was most highly expressed in monocytes, lowest in T cells, and significantly reduced in IBD in monocytes and B cells and, to a lesser extent, neutrophils (Fig. 4 A). The eigengene of the Tan inflammatory response module was elevated only in monocytes, however (Fig. 4 B). Consistent with this, negative correlation between miR-374a-5p expression and the inflammatory response eigengene was stronger in monocytes, weaker in B cells, and absent in other cell types (Fig. 4 C). These data suggest the major regulatory impact of miR-374a-5p on inflammation will be in the monocyte lineage, in both CD and UC.

**Figure S2.**
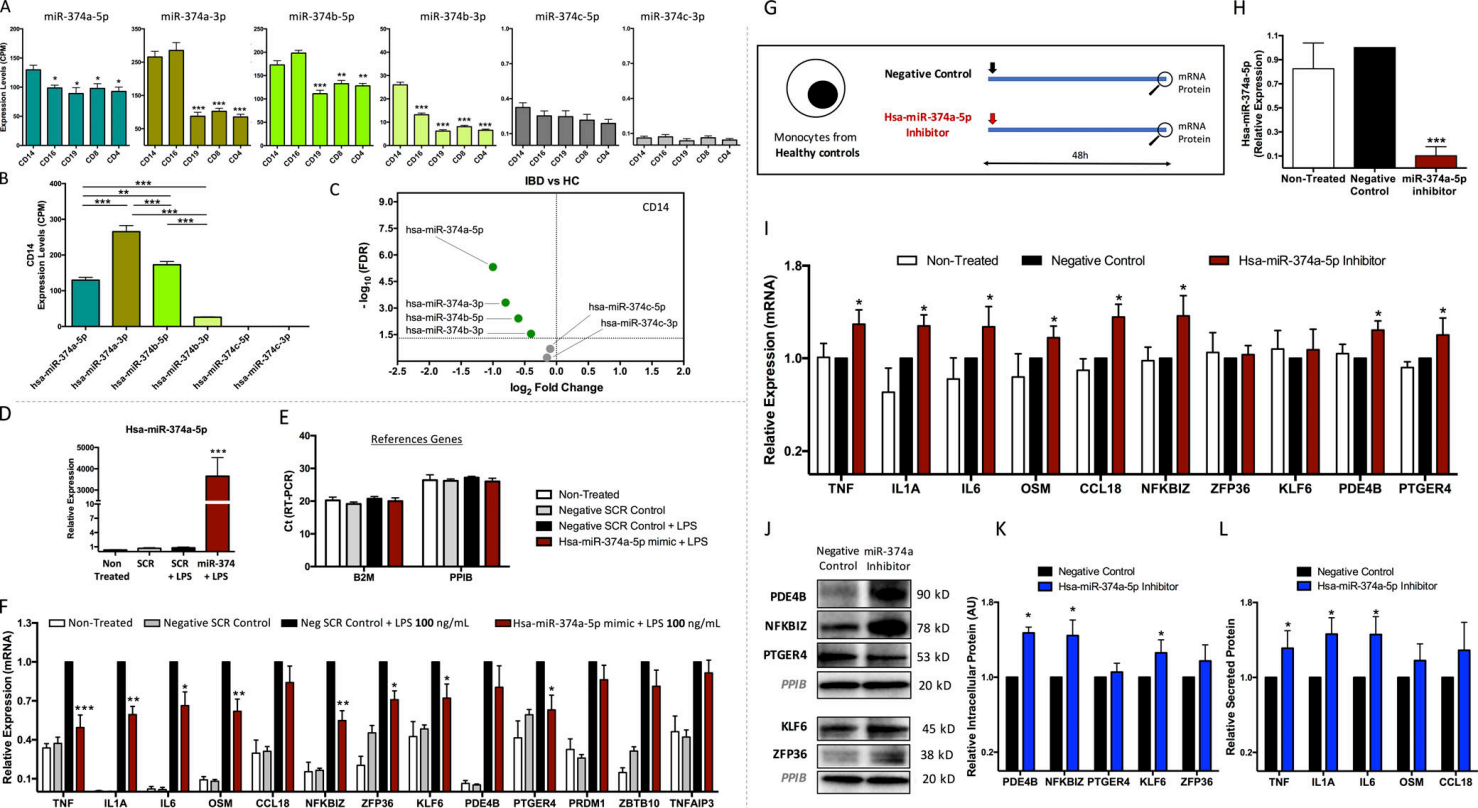
**miR-374a-5p family members and transfection experiments with miR-374a-5p mimic and inhibitor. (A)** Expression of miR-374 family across five immune cell types. *, P < 0.05; **, P < 0.01; ***, P < 0.001 vs. CD14. Unpaired *t* test. **(B)** Comparison of the expression of miR-374 family in monocytes. **, P < 0.01; ***, P < 0.001. One-way ANOVA and Tukey’s post hoc test. **(C)** Volcano plot of miR-374 family members DE between IBD and HC in CD14^+^ monocytes. Green dots, DE miRs down-regulated in IBD; horizontal dashed line, P = 0.05. **(D and E)** Expression level of miR-374a-5p (D) and reference genes (E) after 48 h of transfection of healthy LPS-stimulated monocytes with miR-374a-5p mimic or scrambled control by RT-PCR. **(F)** mRNA expression levels of miR-374a-5p targets after 48 h of mimic transfection of healthy monocytes stimulated with 100 ng/ml of LPS. Data are normalized against negative scrambled control + LPS. Mean and SD of five independent experiments are shown. *, P < 0.05; **, P < 0.01; ***, P < 0.001 vs. SCR control + LPS. Wilcoxon test. **(G)** Experimental design of transfection experiments with 100 nM of miR-374a-5p inhibitor in healthy monocytes for 48 h. **(H)** Expression level of miR-374a-5p after transfection with miR-374a-5p inhibitor or negative control by RT-PCR. **(I)** mRNA expression levels of miR-374a-5p targets after inhibitor transfection. Data were normalized against the negative control. Mean and SD of five independent experiments are shown. **(J)** Western blot analysis of intracellular miR-374a-5p targets. **(K)** Densitometric analysis of Western blot experiments. Mean and SD of four independent experiments are shown. **(L)** Protein levels of secreted miR-374a-5p targets by Luminex assay. Mean and SD of five independent experiments are shown. *, P < 0.05; **, P < 0.01; ***, P < 0.001 vs. negative control. Wilcoxon test.

Along with miR-374a-5p, other members of the miR-374 family were also down-regulated in monocytes from IBD patients (Table S2). We extended this observation, analyzing the miR-374 family across five cell types. Expression of four members of the family (miR-374a-5p, miR-374a-3p, miR-374b-5p, and miR-374b-3p) was seen across immune cell subsets, was prominent in monocytes, and was down-regulated in patients with active IBD in this cell type. The most prominent fall in terms of both magnitude and statistical significance was in miR-374a-5p (Fig. S2, A–C).

### Confirmation of the disease association of miR-374a-5p/inflammatory module in intestinal biopsies

If the miR/mRNA disease associations we see in circulating monocytes are important in disease pathogenesis, they might also be detectable in diseased tissue. We therefore explored this in external published transcriptomic datasets from intestinal biopsies, both extending our findings to the tissue level and providing further validation in independently recruited cohorts.

**Figure 5.**
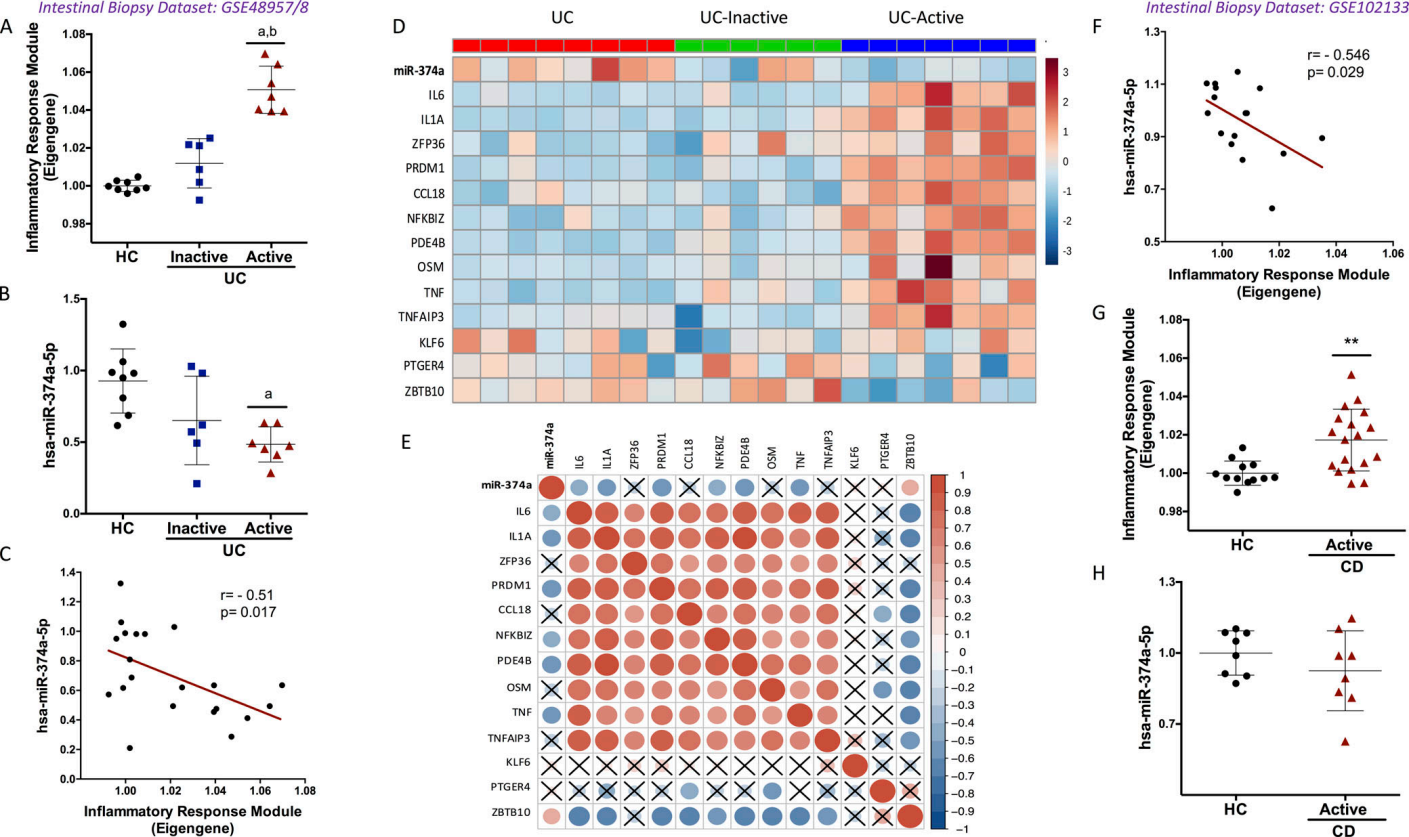
**Correlation of miR-374a-5p and the inflammatory response module in IBD intestinal biopsies. (A and B)** Inflammatory response module eigengene (A) and miR-374a-5p expression (B) in intestinal biopsies of UC patients with differential disease activity. ^a^, P < 0.05 vs. HC; ^b^, P < 0.05 vs. UC-inactive. Kruskal–Wallis and Dunn’s post hoc test (eigengene values normalized to HC). **(C)** Correlation of inflammatory response module eigengene and miR-374a-5p expression in UC biopsies. **(D and E)** Heatmap of expression (D) and correlations of miR-374a-5p/targets (E) in UC biopsies. Red and blue represent high and low normalized expression, respectively, in D and positive and negative correlations in E. Dot size indicates magnitude of the correlation coefficient. X, P > 0.05. Pearson’s correlation test. **(F)** Correlation of inflammatory response module eigengene and miR-374a-5p expression in CD biopsies. **(G and H)** Inflammatory response module eigengene (G) and miR-374a-5p expression (H) in ileal biopsies of active CD patients. **, P < 0.01 vs. HC. Unpaired *t* test (eigengene values normalized to HC; H).

A dataset from biopsies of seven active and six inactive UCs and eight HCs was first analyzed (GSE48957, GSE48958; Van Der Goten et al., 2014) and showed elevation of the inflammatory response module eigengene and reduced miR-374a-5p expression in active UC patients compared with inactive patients and HC (Fig. 5, A and B). The negative correlation seen between the two (Fig. 5 C) was comparable to that observed in peripheral blood (Fig. 4 C). Consistent with this, the expression of most potential targets of miR-374a-5p within the module was up-regulated in patients with active UC (Fig. 5 D), and a negative correlation between miR-374a-5p and some of its targets (*IL6*, *IL1A*, *PRDM1*, *NFKBIZ*, *PDE4B*, and *TNF*) was seen (Fig. 5 E).

The significant inverse correlation between the expression of miR-374a-5p and the inflammatory module was validated in a second dataset of ileal mucosal biopsies from 12 HC and 18 active and newly diagnosed CD patients (GSE102133; Verstockt et al., 2019; Fig. 5 F). In this dataset, the inflammatory module was significantly up-regulated, and miR-374a-5p was slightly down-regulated compared with HC (Fig. 5, G and H).

**Figure 6.**
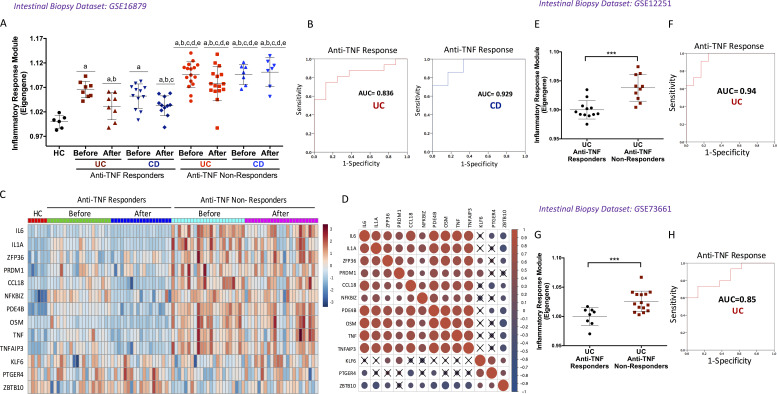
**The inflammatory response module predicts response to anti-TNF therapy in IBD intestinal biopsies. (A)** Inflammatory response module eigengene values in intestinal biopsies of HC and UC and CD patients before and after 4–6 wk of anti-TNF therapy. ^a^, P < 0.05 vs. HC; ^b^, P < 0.05 vs. UC anti-TNF responder before therapy; ^c^, P < 0.05 vs. CD anti-TNF responder before therapy; ^d^, P < 0.05 vs. UC anti-TNF responder after therapy; ^e^, P < 0.05 vs. CD anti-TNF responder after therapy. Kruskal–Wallis and Dunn’s post hoc test. **(B)** Receiver operator characteristic curve using the inflammatory response module eigengene before therapy as a predictor of anti-TNF response in UC and CD. **(C and D)** Heatmap of the expression (C) and correlation of miR-374a-5p targets (D) in UC and CD biopsies before and after anti-TNF therapy. Red and blue represent high and low normalized expression, respectively, in C and positive and negative correlations in D. Dot size indicates magnitude of the correlation coefficient. X, P > 0.05. Pearson’s correlation test. **(E–H)** Validation of the inflammatory module eigengene as predictor of anti-TNF response in two independent datasets of intestinal UC biopsies. ***, P < 0.001. Unpaired *t* test.

A third dataset compared active UC and CD treated with anti-TNF therapy with HC (GSE16879; Arijs et al., 2009a). The inflammatory response module eigengene was higher in both active UC and CD patients. Intriguingly, an increased eigengene value in biopsies taken before therapy was highly predictive of subsequent nonresponse to anti-TNF therapy (area under the curve [AUC] = 0.83 and AUC = 0.92 in UC and CD patients respectively; Fig. 6, A and B). Consistent with this, up-regulation of most of the potential targets of miR-374a-5p was observed in both UC and CD nonresponders (Fig. 6 C), and their correlated expression confirmed (Fig. 6 D). The inflammatory response module eigengene as predictor of anti-TNF response was validated in two independent datasets (GSE12251 [Arijs et al., 2009b] and GSE73661 [Arijs et al., 2018]) of intestinal biopsies from UC patients (Fig. 6, E–H).

### In vitro exploration of the interaction between miR-374a-5p and its proinflammatory targets

**Figure 7.**
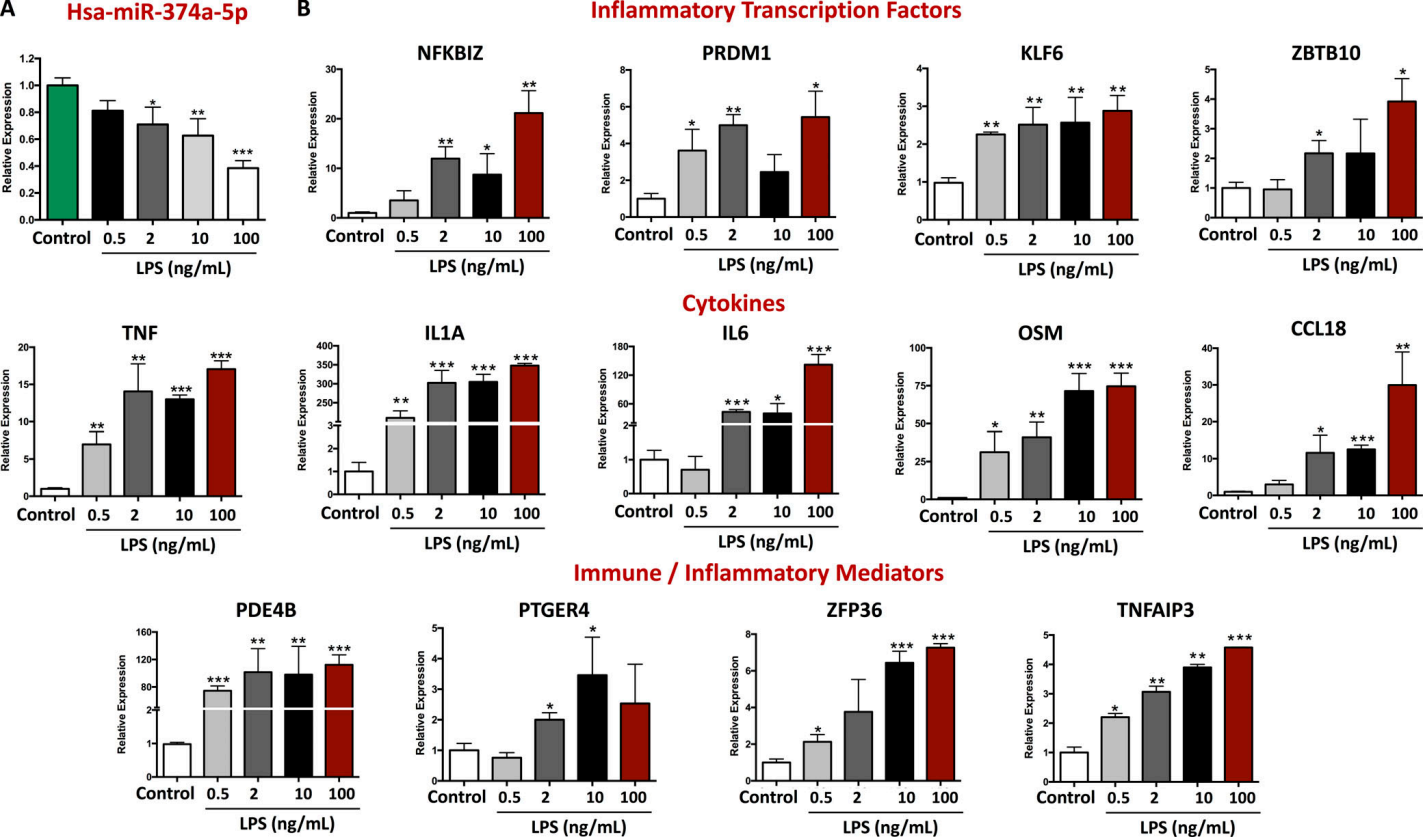
**LPS stimulation of miR-374a-5p and its proinflammatory targets.** Monocytes from HC were activated with different doses of LPS (0.5, 2, 10, and 100 ng/ml) for 24 h, and the expression levels of miR-374a-5p and its targets were evaluated by RT-PCR. **(A)** Expression level of miR-374a-5p. **(B)** Expression level of potential miR-374a-5p targets. Mean and SD are represented. *, P < 0.05; **, P < 0.01; ***, P < 0.001 vs. control. Mann–Whitney *U* test.

The correlation between miR-374-5p and inflammatory gene expression supports a role for miR-374-5p in monocyte physiology in IBD, but correlation does not prove causation. To directly investigate the role of miR-374a-5p in monocyte biology, we examined the expression of miR-374a-5p and its targets after monocyte stimulation using LPS. This is a relevant model for IBD, since it has been well established that the aberrant immune response is directed toward components of the intestinal microflora (Zhang et al., 2017), and the Tan module not only contained genes involved in inflammation, but was also enriched for features of the response to LPS (Fig. S1 B). Consistent with a direct role for miR-374a-5p during inflammatory responses, its expression was reduced (Fig. 7 A), and that of its targets increased (Fig. 7 B), by LPS in a dose-dependent manner.

**Figure 8.**
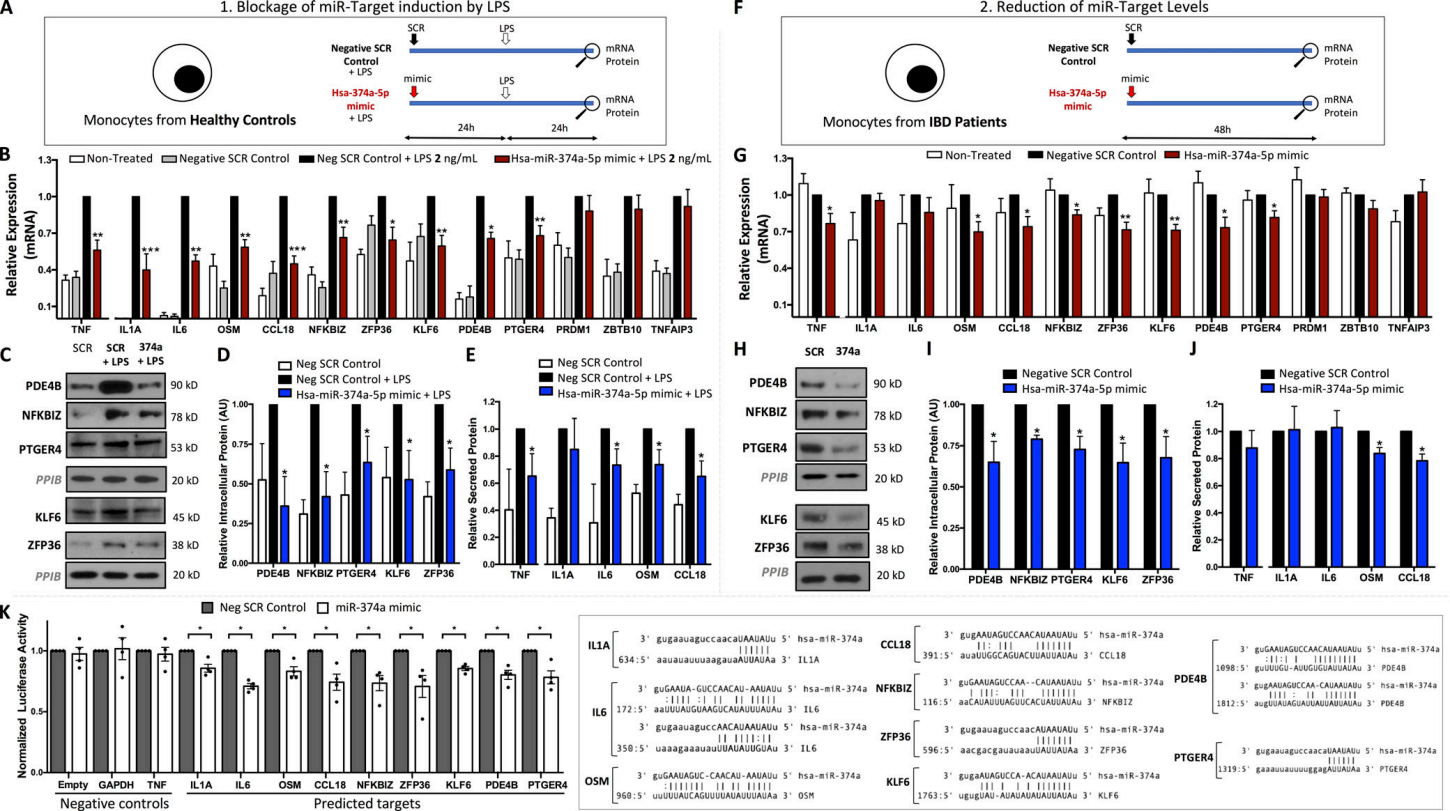
**In vitro validation of coexpressed proinflammatory genes as targets of miR-374a-5p. (A)** Experimental design of transfection experiments with 100 nM of miR-374a-5p mimic in healthy monocytes stimulated with 2 ng/ml of LPS. **(B)** mRNA expression levels of miR-374a-5p targets after mimic transfection. Data were normalized against a negative scrambled control + LPS. Mean and SD of five independent experiments are represented. **(C)** Western blot analysis of intracellular miR-374a-5p targets. **(D)** Densitometric analysis of Western blot experiments. Mean and SD of three independent experiments are shown. **(E)** Protein levels of secreted miR-374a-5p targets by Luminex assay. Mean and SD of five independent experiments are shown. **(F)** Experimental design of transfection experiments with 100 nM of miR-374a-5p mimic in monocytes from IBD patients. **(G)** mRNA expression levels of miR-374a-5p targets after mimic transfection. Data were normalized against a negative scrambled control. Mean and SD of five independent experiments are represented. **(H)** Western blot analysis of intracellular miR-374a-5p targets. **(I)** Densitometric analysis of Western blot experiments. Mean and SD of three independent experiments are shown. **(J)** Protein levels of secreted miR-374a-5p targets by Luminex assay. Mean and SD of five independent experiments are shown. *, P < 0.05; **, P < 0.01; ***, P < 0.001 vs. SCR control + LPS (A–E) or vs. SCR control (F–J). Wilcoxon test. **(K)** Luciferase activity of HEK293 cells cotransfected with SwithGear GoClone reporter constructs and either negative scrambled control or miR-374a-5p mimic. Data were normalized against negative scrambled control. Mean and SD of four independent experiments are shown. *, P < 0.05 vs. SCR control. Wilcoxon test.

To further confirm the ability of miR-374a-5p to directly control expression of its inflammatory targets, monocytes from healthy individuals were transfected with a miR-374a-5p mimic; 24 h later, they were stimulated with 2 ng/ml of LPS for a further 24 h (Fig. 8 A). Increased miR-374a-5p expression after transfection was confirmed by RT-PCR (Fig. S2 D). LPS induced all predicted targets, and pre-transfection with a miR-374a-5p mimic reduced the induction of *TNFα*, *IL1A*, *IL6*, *OSM*, *CCL18*, *NFKBIZ*, *ZFP36*, *KLF6*, *PDE4B*, and *PTGER4* consistent with a direct regulatory effect (Fig. 8 B). This effect was also seen, though was less pronounced, when supraphysiological doses of LPS were used (100 ng/ml; Fig. S2, E and F). miR-374a-5p inhibition of these targets was confirmed at protein level in the same samples: intracellular proteins (*PDE4B*, *NFKBIZ*, *PTGER4*, *KLF6*, and *ZFP36*) by Western blot and secreted proteins (*TNF*, *IL6*, *OSM*, and *CLL18*) by Luminex assays of culture supernatants (Fig. 8, C–E).

To complement the analysis of in vitro–stimulated HC monocytes, we studied the capacity of miR-374a-5p to reduce its proinflammatory targets in monocytes from patients with active IBD immediately ex vivo. Transfection with a miR-374a-5p mimic reduced *TNFα*, *OSM*, *CCL18*, *NFKBIZ*, *ZFP36*, *KLF6*, *PDE4B*, and *PTGER4* mRNA compared with the negative scrambled control, again supporting a direct regulatory role and revealing a potential therapeutic opportunity (Fig. 8, F and G). This was again confirmed at a protein level by Western blot (*PDE4B*, *NFKBIZ*, *PTGER4*, *KLF6*, and *ZFP36*) or supernatant assay (*OSM* and *CCL18*) in the same experiments (Fig. 8, H–J). Luciferase assays were used to evaluate the direct interaction between the seed sequence of miR-374a-5p and the 3-UTR region of those coexpressed predicted targets reduced by the miR-374a-5p mimic. Such interaction was confirmed for *IL1A*, *IL6*, *OSM*, *CCL18*, *NFKBIZ*, *ZFP36*, *KLF6*, *PDE4B*, and *PTGER4*, but not *TNFα* (Fig. 8 K).

Because overexpression experiments with mimics are likely to provide a supraphysiological level of miRNA, it was important to confirm that endogenous miR-374a-5p has a functional impact. We inhibited miR-374a-5p with a sequence-specific miRNA antagonist in healthy monocytes (Fig. S2 G). After 48 h, the level of miR-374a-5p was reduced (Fig. S2 H). In parallel, the expression of most of the predicted targets of miR-374a-5p was significantly up-regulated at the mRNA level (*TNF*, *IL1A*, *IL6*, *OSM*, *CCL18*, *NFKBIZ*, *PDE4B*, and *PTGER4*) or/and protein level (*PDE4B*, *NFKBIZ*, *KLF6*, *TNF*, *IL1A*, and *IL6*; Fig. S2, I–L).

**Figure S3.**
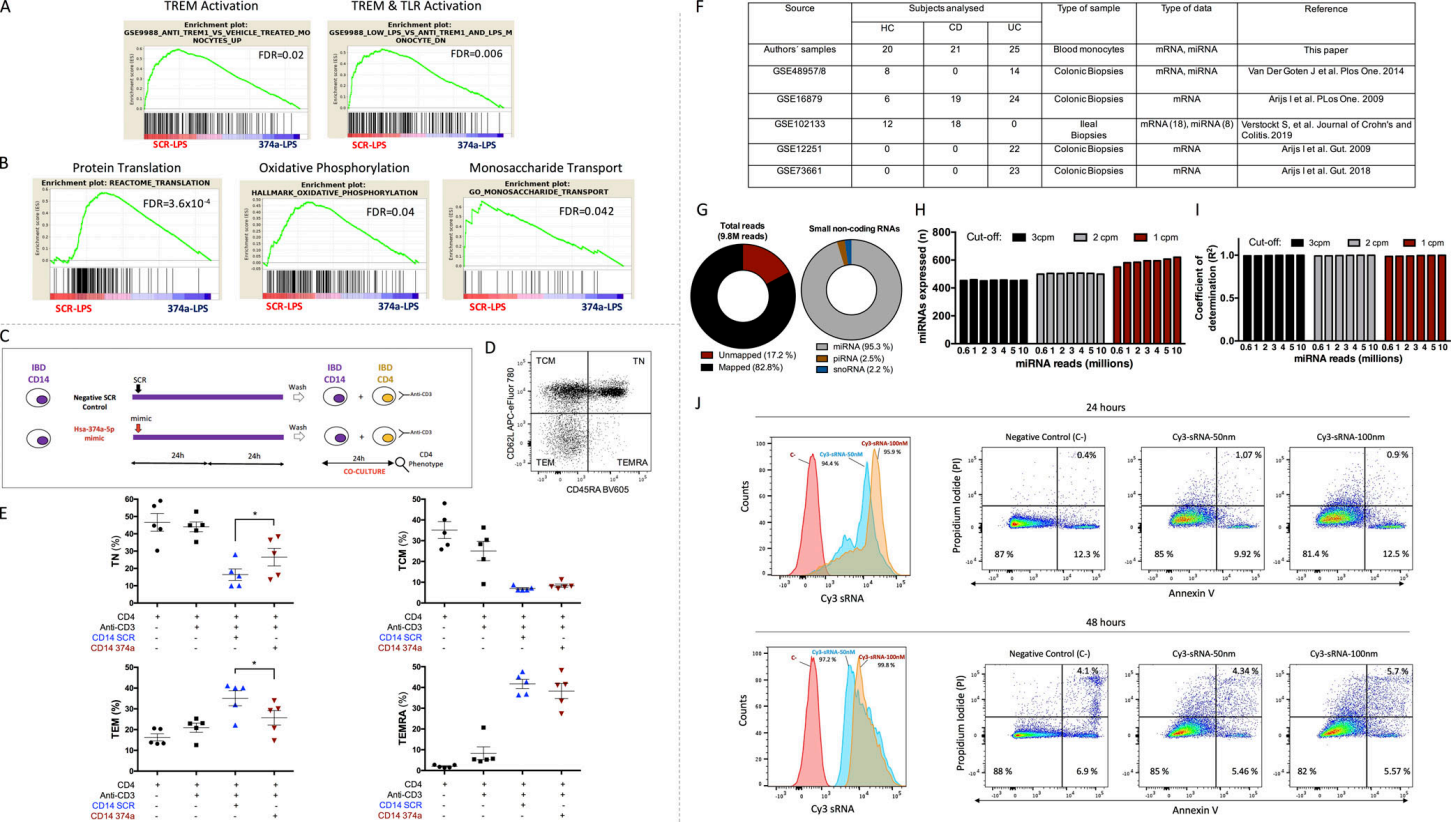
**Global transcriptional impact of miR-374a-5p overexpression, co-culture experiments of CD4 T cells, and monocytes from IBD patients and quality control data on miRNA datasets. (A and B)** RNA-seq and GSEA of miR mimic transfections identified a global impact of miR-374a-5p in cell activation pathways. RNA-seq was performed on RNA from transfection experiments of LPS-stimulated healthy monocytes with SCR control or miR-374a-5p mimic. GSEA identified the top enriched set of genes between the two conditions: (A) anti-TREM1 vs. vehicle-treated monocytes up-regulated genes; low LPS vs. anti-TREM1 and LPS-treated monocytes down-regulated genes; and (B) translation, oxidative phosphorylation, and monosaccharide transport pathways. **(C)** Experimental design chart of coculture experiments between anti-CD3–stimulated CD4 T cells and autologous monocytes pretransfected with SCR or miR-374a-5p mimic from IBD patients. **(D)** Representative dot plot showing the differentiation status of CD4^+^ T cells from IBD before coculture. **(E)** The percentages of T_CM_, T_N_, T_EM_, and T_EMRA_ were analyzed by flow cytometry after 24 h of coculture. *, P < 0.05. **(F)** Summary of the mRNA and miRNA dataset used in this study. **(G)** Description of the mapping results of the small-RNA-seq analysis. **(H and I)** Expressed miRNAs (H) and coefficient of determination (I) of the subsampling small RNA-seq analysis. **(J)** Transfection efficiency and cell viability of miR mimic experiments. Fluorescent Cy3-negative scrambled control was transfected into healthy monocytes for 24 h (above) and 48 h (below) at 50 and 100 nM, and both transfection efficiency (left) and cell viability (right) were assessed by flow cytometry. Alive cells: PI^−^, Annexin V^−^; early apoptotic cells: PI^−^, Annexin V+; late apoptotic cells: PI^+^, Annexin V^+^.

We next sought to define the impact of miR-374a-5p on monocyte transcription, to determine if it extended beyond the genes identified in the Tan module. We therefore transfected healthy monocytes with SCR or miR-374a-5p mimic before LPS activation and performed gene set enrichment analysis (GSEA) on RNA-seq data generated from them. Enrichments of known LPS- and TREM-response genes (Dower et al., 2008; Fig. S3 A) were identified. The expression of leading-edge genes (that drive the enrichment) was reduced by pretransfection with an miR-374a-5p mimic. These included genes related to leukocyte migration (*CSF1*, *CCL20*, *F3*, *LGALS3*, *SPRY2*, *PTGS2*, and *DAB2*), PPAR signaling (*PPARG*, *MMP1*, *LPL*, and *FABP5*), TNF signaling (*TNFSF15*, *TNFSF14*, *EDN*, and *PTGS2*), and innate immunity (*AKIRIN2*, *CRTAM*, *DDIT3*, *EIF2AK3*, *INHBA*, *LY9*, *PRGS2*, *RAB20*, *RAB7B*, *RABTB*, *S1PR3*, and *SIGLEC15*). In addition, canonical pathways associated with cellular activation, such as protein translation, oxidative phosphorylation and monosaccharide transport, were enriched after transfection, with their leading-edge genes also down-regulated by a miR-374a-5p mimic (Fig. S3 B). Approximately 19% of the down-regulated genes showed complementarity with the seed sequence of miR-374a-5p, but this enrichment did not reach statistical significance. This observation is in keeping with previous studies that report that ∼20–25% of differentially regulated transcripts are predicted targets of a transfected miRNA (Shahab et al., 2012; Matkovich et al., 2012; Jones et al., 2015; Thomas et al., 2010).

### Functional studies support miR-374a-5p as a key regulator of monocyte-driven inflammation

**Figure 9.**
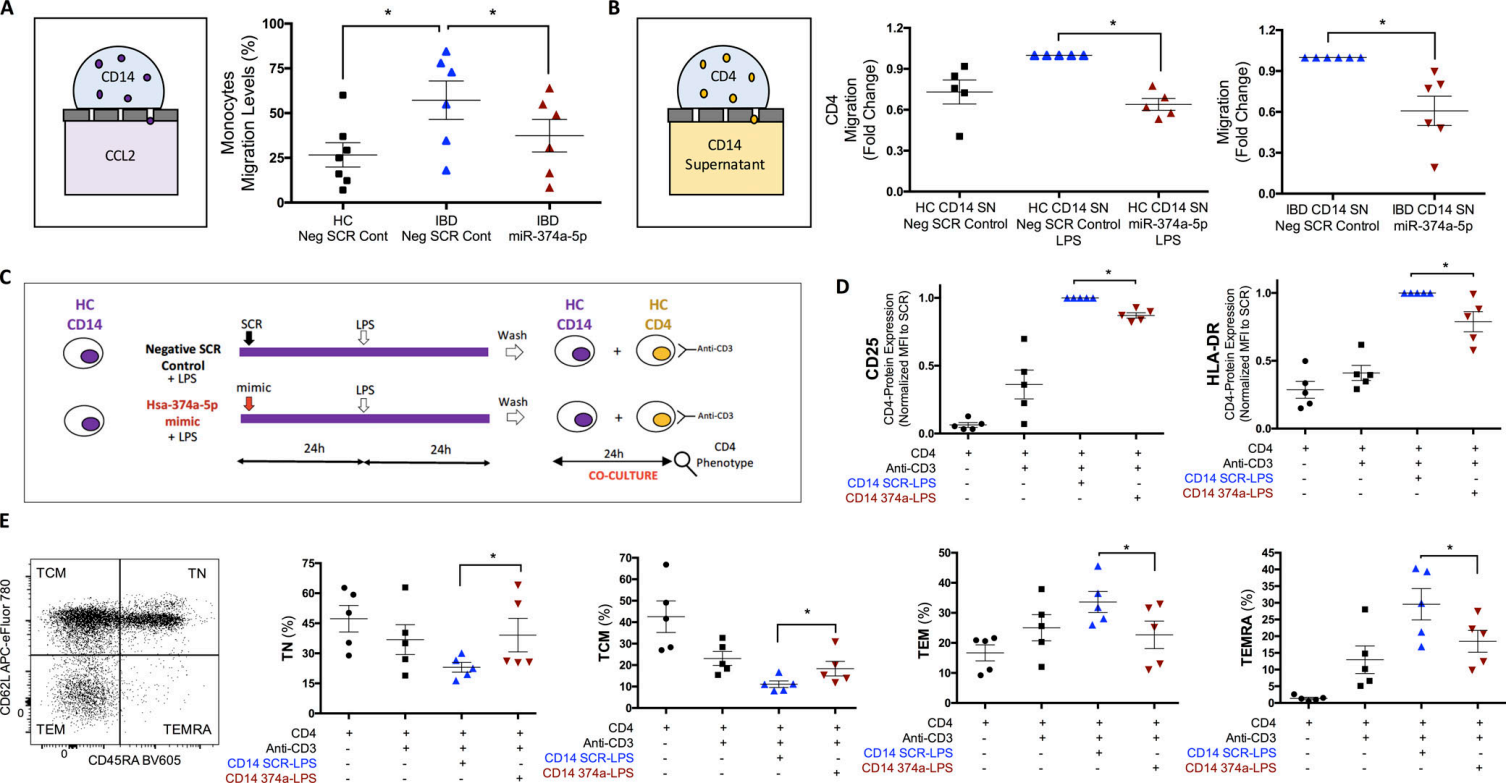
**miR-374a-5p regulated key monocyte functions such as migration and T-cell activation. (A)** Migration assays of monocytes from seven HC and six IBD patients transfected with SCR or miR-374a-5p mimic. Cells were monitored with Calcein, and a ChemoTx Disposable Chemotaxis System was used, with CCL2 as a chemoattractant. Mean and SD are shown. **(B)** Migration assays of CD4^+^ T cells, using as chemoattractant the supernatant of monocytes transfected with SCR or miR-374a-5p mimic from LPS-stimulated HC (left) or IBD patients (right). **(C)** Experimental design chart of coculture experiments between anti-CD3–stimulated CD4^+^ T cells and autologous LPS-stimulated monocytes pretransfected with SCR or miR-374a-5p mimic. **(D)** T cell activation markers (CD25 and HLA-DR) evaluated by flow cytometry on CD4^+^ T cells after coculture. **(E)** Representative dot plot showing the differentiation status of CD4^+^ T cells from HC before coculture (left). The percentages of T_CM_, T_N_, T_EM_, and T_EMRA_ were analyzed by flow cytometry after 24 h of coculture (right). Mean and SD of five independent experiments are shown. SCR, scrambled control; T_CM_, CD45RA^−^CD62L^+^; T_N_, CD45RA^+^CD62L^+^; T_EM_, CD45RA^−^CD62L^−^; T_EMRA_, CD45RA^+^CD62L^−^. *, P < 0.05. Wilcoxon test. Mann–Whitney *U* test was used in A between HC and IBD.

miR-374a-5p, by directly targeting specific inflammatory targets, suppresses many of the mRNA pathways that underpin monocyte responses to inflammatory stimuli. We thus sought to determine if this had an impact on monocyte function after activation. We evaluated the role of miR-374a-5p on the migration capacity of monocytes using a *CCL2* chemotaxis assay. Monocytes from active IBD patients showed an increased migration capacity towards *CCL2*, which was reduced to a level similar to that of HC monocytes by transfection with a miR-374a-5p mimic (Fig. 9 A).

Monocyte-derived macrophages are also thought to drive inflammation in IBD by attracting and activating other immune cells, including CD4^+^ T cells (Podolsky, 2002; Neurath, 2019). We thus examined the impact of miR-374a-5p mimic transfection on the ability of monocytes to attract, activate, and differentiate CD4^+^ T cells. Pretransfection with miR-374a-5p reduced monocyte-driven CD4^+^ T cell chemoattractant capacity when either LPS-stimulated HC monocytes or unstimulated monocytes from patients with active IBD were used (Fig. 9 B). The effect of miR-374a-5p transfection on CD4^+^ T cell phenotype was also evaluated in cocultures (Schrier et al., 2016). LPS-stimulated monocytes from HC transfected with either miR-374a-5p and negative scrambled control were cocultured with autologous CD4^+^ T cells prestimulated with anti-CD3, and activation markers (*CD25*, *HLA-DR*), and proportions of naive (T_N_) and memory CD4^+^ T cell subsets (central memory [T_CM_], effector memory [T_EM_], and terminally differentiated effector memory [T_EMRA_]) were analyzed. Monocytes transfected with miR-374a-5p reduced the expression of CD25 and HLA-DR in CD4^+^ T cells and reduced T effector differentiation (reducing the increase in T_EM_ and T_EMRA_ subsets; Fig. 9, C–E). A similar, though less pronounced, trend was observed when autologous CD4^+^ T cells from active IBD patients were co-cultured with IBD monocytes transfected with miR-374a-5p (Fig. S3, C–E). Altogether, this demonstrates that miR-374a-5p is a critical regulator of many facets of the monocyte inflammatory response and highlights the therapeutic potential of targeting this miR in IBD.

## Discussion

Monocytes are thought to be central players in IBD, as the source of the monocyte-derived macrophages that drive inflammation (Na et al., 2019; Baillie et al., 2017; Podolsky, 2002; Neurath, 2019). Using integrative mRNA and miRNA analysis in monocytes from IBD patients, we identified miR-374a-5p as a potential central regulator in IBD, as its down-regulation increases expression of a module of key inflammatory mediators. We showed that the overexpression of miR-374a-5p turned off both inflammatory functions of IBD monocytes and their impact on CD4^+^ T cells, and in doing so, identified a previously unrecognized role for miR-374a-5p in the pathogenesis of immune-mediated disease, in particular IBD. In addition to the prominent association between miR-374a-5p expression and IBD, other members of the miR-374 family were similarly, if less strikingly, associated, raising the possibility that they may be controlled by similar upstream mechanisms as miR-374a-5p, and may also have a functional impact on disease.

Previous studies have associated changes in miR-374a-5p expression with a range of diseases or disease models, including neonatal hypoxic brain damage (Chen et al., 2020; Looney et al., 2015), ischemia/reperfusion injury (Huang et al., 2019), osteoarthritis (Shi and Ren, 2020), and obesity (Doumatey et al., 2018). While these studies did not explore the role of miR-374a-5p in immune cells, an impact on inflammation is suggested by some of the predicted target genes described (e.g., *NLRP3* [Chen et al., 2020], *Smad6* [Chen et al., 2020], *CCL2* [Doumatey et al., 2018], JNK signaling [Gong et al., 2018]). miR-374a-5p is also seen in lists of down-regulated miRNAs in profiling studies in immune-mediated diseases, including SLE (Luo et al., 2013; Pérez-Sánchez et al., 2016; Van Den Hoogen et al., 2018), rheumatoid arthritis (Arias de la Rosa et al., 2020), antiphospholipid syndrome (Pérez-Sánchez et al., 2018), Sjögren’s syndrome (Wang-Renault et al., 2018), axial spondyloarthritis (Prajzlerová et al., 2017), asthma (Williams et al., 2009), and diabetes (Stępień et al., 2018; Yang et al., 2018). These studies did not specifically mention miR-374a-5p and did not provide functional evidence for a role in pathogenesis.

miR-374a-5p has not been previously functionally implicated in the pathogenesis of inflammatory disease. miR-374a-5p was inversely correlated with a gene module up-regulated in monocytes from UC and CD (and SLE) patients, and in IBD intestinal biopsies module expression was associated with disease activity and could predict response to anti-TNF therapy, a finding validated in independent datasets. If confirmed in larger studies, this predictive marker of treatment response could help address a critical unmet need in the field. The Tan module is negatively correlated with miR-374a-5p expression, but while it is enriched for validated targets of miR-374a-5p, many genes in it lack putative seed sequences, and are therefore unlikely to represent direct targets of the miR. It is known that transcription factors and miRs work in parallel to modulate gene expression. Thus, some of the genes in the Tan module may represent indirect targets of miR-374a-5p, regulated by transcription factors that are themselves directly regulated by miR-374a-5p. Additionally, LPS signaling itself leads to a highly coregulated transcriptional response, which includes the up-regulation of many genes found within the Tan module. Thus, a proportion of Tan module genes could be modulated directly by LPS, and thus be coregulated with, but independent of, miR-374a-5p.

The miR-374a-5p–associated module contained a remarkably focused set of known key inflammatory genes (*IL6*, *OSM*, *IL1A*, *CCL18*, *KLF6*, *NFKBIZ*, *ZFP36*, *PDE4B*, *PTGER4*, and *TNF*); all but *TNF* were shown to be direct targets of miR-374a-5p. miR-374a-5p mediates control these canonical pathways, which drive an activated phenotype in monocytes including an increased migration capacity, and increased activation of CD4^+^ T-cells. Given the inflammatory importance of genes controlled by miR-374, at least four of which are targets of existing drugs, the identification of other disease-associated miRNAs could lead us to new downstream inflammatory mediators, or upstream regulators of the miRNAs that might be therapeutically targeted.

miRNAs are attractive as novel therapeutics in themselves, with the field’s main challenge being the develop of effective miRNA tissue or cell-specific delivery systems (Li and Rana, 2014; Rupaimoole et al., 2011). Nonetheless Miravirsen, an anti–miR-122 reached phase II clinical trials for hepatitis C virus treatment, and an miR-34 mimic MRX34 is in phase I clinical trials to treat different solid tumors (Rupaimoole and Slack, 2017). An alternative strategy is to discover what regulates miRNA production, which might reveal more tractable therapeutic targets (Agostini and Knight, 2014).

Our data suggest miR-374a-5p has considerable therapeutic potential. In vivo preclinical studies are limited by the absence of a mouse ortholog (miR-374a-5p is conserved in the Rhesus macaque, pig, goat, armadillo, rabbit, and shrew: https://www.mirbase.org/). Issues with therapeutic delivery would also arise, but topical therapy might be possible in UC, where inflammation is predominantly mucosal and restricted to the colon. miRNA delivery to the liver is relatively straightforward—for example Patisiran, an siRNA that silences transthyretin in hereditary transthyretin-mediated amyloidosis, is directly delivered to the liver and is the first Food and Drug Administration–approved RNA-based therapy (Heras-Palou, 2019). This raises the possibility that miR-374a-5p could be used to treat liver diseases in which miR-374a-5p is reduced, such as non-alcoholic steatohepatitis; miR-374a-5p reduction is associated with progressive fibrosis; Estep et al., 2010), hepatitis C virus (Zhang et al., 2015), and or progressive fibrosis in hepatitis B (Bao et al., 2017). Taken together, our studies identify miR-374a-5p as a potential master regulator of monocyte-driven inflammation in IBD, which may have clinical implications in a range of immune-mediated diseases.

## Materials and methods

### Subjects

46 patients with active IBD (25 with UC and 21 with CD, diagnosed using standard criteria; Silverberg et al., 2005) were recruited before starting treatment at a specialist IBD clinic at Addenbrooke’s Hospital in Cambridge, UK. Assessment of disease activity was in accordance with national (British Society of Gastroenterology) and international guidelines (European Crohn’s and Colitis Organisation; Walsh et al., 2016). Sigmoidoscopy or colonoscopy was performed where appropriate. Harvey–Bradshaw severity index (<4 remission) or simple clinical colitis activity index (<2 remission) was assessed for CD and UC, respectively (Table S1). In parallel, a cohort was recruited of 20 age-matched adult HC volunteers with no family history of autoimmune disease, no serious comorbidities, no use of steroids or immunosuppressants, and no hospitalization within the last 12 mo.

64 patients with active AAV and 49 patients with active SLE were recruited before starting treatment at Addenbrooke’s Hospital. AAV included granulomatosis with polyangiitis (formerly Wegener’s granulomatosis) and microscopic polyangiitis; eosinophilic granulomatosis with polyangiitis (formerly Churg–Strauss syndrome) was excluded. Active disease was defined by the Birmingham Vasculitis Activity Score (Stone et al., 2001). SLE patients met at least four of the American College of Rheumatology SLE criteria (Tan et al., 1982). Active disease was defined as meeting all the following defined criteria: new British Isles Lupus Assessment Group score A or B in any system (Isenberg et al., 2005), clinical assessment of active disease by the reviewing specialist, and increase in immunosuppressive therapy as a result.

### Cell separation

PBMCs from IBD, AAV, SLE, and HC were isolated from blood samples (100 ml) using Histopaque 1077 (Sigma-Aldrich) and separated into two different fractions. CD14^+^ monocytes and CD19^+^ B cells were isolated from each fraction by positive immunomagnetic selection (Miltenyi Biotec; Lyons et al., 2007). CD4^+^ and CD8^+^ T cells were isolated by positive selection from the CD14^−^ and CD19^−^ fractions, respectively. Finally, CD16^+^ cells were isolated by red cell lysis from the red cell/granulocyte pellet, followed by CD16 positive selection. For cell migration experiments, monocytes were isolated from PBMCs by negative selection using the Pan Monocyte Isolation Kit (Miltenyi Biotec).

### RNA extraction, microarray preprocessing, and differential expression analysis

RNA was extracted from CD14^+^, CD16^+^, CD19^+^, CD8^+^, and CD4^+^ cells from all the subjects included in the study using the RNEasy Plus Mini Kit (Qiagen) according to the manufacturer’s instructions. RNA quantity and integrity were determined by NanoDrop and Agilent 2100 Bioanalyzer (Agilent Technologies). 200 ng of RNA was processed for hybridization onto Affymetrix Human Gene ST 1.1 microarrays, according to the manufacturer’s instructions. Background correction, quantile normalization, and log_2_ transformation were performed using the robust multiarray average method in R (v3.6.0). Samples were subjected to quality control evaluation and batch effect correction if needed. Probe annotation and differential expression were obtained from the pd.hugene.1.1.st.v1 and limma packages, respectively. An FDR < 0.05 was considered significant after multiple correction testing.

Five different datasets from biopsy samples taken from diseased colon of IBD patients were also analyzed from the Gene Expression Omnibus (GEO). The GEO dataset GSE48957/GSE49958 included mRNA and miR paired data from mucosal biopsies of 8 HC and 14 UC patients (6 inactive and 8 active patients; Van Der Goten et al., 2014). The dataset dataset GSE16879 included mRNA data of mucosal biopsies from 6 HC and 43 IBD patients (19 CD and 24 UC) before and after anti-TNF therapy (infliximab). Patients were considered responders or nonresponders based on endoscopic and histologic findings at the end of the treatment (8 wk; Arijs et al., 2009a). The dataset dataset GSE102133 contained paired mRNA and miRNA data from ileal mucosal biopsies of 12 HC and 18 active and newly diagnosed CD patients (Verstockt et al., 2019). The GEO dataset GSE12251 included mRNA data of mucosal biopsies from 22 UC patients before infliximab treatment (12 responders and 10 nonresponders). Response to anti-TNF was defined as endoscopic and histologic healing at week 8 (Arijs et al., 2009b). Finally, the GEO dataset GSE73661 contained mRNA data from colonic biopsies of 22 UC patients before infliximab therapy (8 responders and 15 nonresponders). Response to anti-TNF was defined as endoscopic and histologic healing at week 4–6 (Arijs et al., 2018). The details of the cohorts used in this study are summarized in Fig. S3 F.

### WGCNA

WGCNA was performed on the microarray data of HC and IBD patients to identify modules of highly coexpressed genes associated with the disease (Langfelder and Horvath, 2008). WGCNA R software package v1.69 was used. The minimum number of genes in a module was set to 30. Each module containing a cluster of highly coexpressed genes was summarized by a representative eigengene profile (first principal component), which was then correlated against a matrix of clinical variables. Modules showing a significant correlation (FDR < 0.05) with disease were selected for further analysis. When different diseases or cell types were compared, raw intensity matrices were normalized together and batch corrected. Pathway enrichment analysis included a network-based protein–protein interaction analysis, STRING, and Gene Ontology enrichment analysis.

### Module preservation assessment

Module preservation assessment was performed with the R WGCNA package to investigate whether given modules in the reference data set (CD14 IBD-HC) could be found in a test data set. These included CD14^+^ AAV-HC, CD14^+^ SLE-HC, CD16^+^ IBD-HC, CD19^+^ IBD-HC, CD8^+^ IBD-HC, and CD4^+^ IBD-HC. The Zsummary statistic was used to assess the overall significance of module preservation (Langfelder et al., 2011).

### Small RNA isolation, sequencing, and analysis

Small RNA was extracted from cell lysates using the RNEasy Plus Mini Kit and the RNeasy MinElute Cleanup Kit (both from Qiagen) according to the manufacturer’s instructions. Briefly, the workflow for small RNA-seq is divided into three main sections: (1) library preparation using the TruSeq small RNA kit from Illumina; (2) preprocessing of fastq files, quality control, and mapping; and (3) downstream analysis. Libraries were sequenced using a NextSeq500 High Output sequencer in single-read, 75-bp mode, to a depth of 9.8 ± 0.6 million reads, of which 82.8% (8.2 ± 0.5 million reads) could be uniquely mapped to the human genome (GRCh 38 build). Of the mapped reads, 95.3% mapped to annotated miRNAs (miRbase v21), 2.5% to piRNAs (piRNAbank v2), and 2.2% to snoRNAs (Ensembl v84; Fig. S3 G).

A threshold of 2 counts per million (cpm) reads was used to determine whether an miRNA was expressed or not in each library. This threshold was selected based on a subsampling study in which a sample containing ∼10 million miRNA reads was randomly sampled at different sequencing depths in triplicate to create datasets containing 0.6, 1, 2, 3, 4, and 5 million miRNA reads. Similar numbers of expressed miRNAs were obtained across all subsampled libraries when either 2 (451–459) or 3 (499–506) cpm was used as the cutoff value of miRNA expression (Fig. S3 H); whereas using a 1-cpm cutoff showed the highest variability in numbers of miRNA deemed expressed (550–620). Furthermore, we found that the expression levels of individual miRNAs were highly correlated between all subsampled libraries and the original library (*R*^2^ = 0.98–0.99; Fig. S3 I). Normalization and differential expression analysis between groups of interest was carried out with the R package DESeq2. miRs showing an FDR < 0.05 were considered DE.

### Integrated mRNA-miR analysis

A novel integrated mRNA-miR analysis was performed on data from CD14^+^ monocytes with the aim of identifying miRs that could regulate modules of highly coexpressed genes associated with IBD. This analysis included two steps. In the first step, a permutation test was performed to identify those miRs that had a significant negative correlation with a given module of genes associated with IBD. To this end, Pearson correlations between a given DE miR and all the genes that composed a given module were computed, and the average of the correlations was identified as the actual mean correlation. Then, 10,000 random permutations of the phenotype labels (i.e., the sample index) were performed, and the mean correlation of this miR with each permuted dataset formed a set of 10,000 background mean correlations. The nominal P value for this miR-module pair was defined as the empirical quantile of the actual mean correlation in relation to the background mean correlations. The above was performed on all DE miRs for a given module, and miRs with both P value < 0.05 and FDR < 0.25 in the permutation test were selected.

In the second step, the identification of gene targets within the module that potentially could be modulated by the miRs was performed. Thus, the combination of TargetScan (v7.1) and miRanda platforms was used to predict direct interactions. Finally, potential miR-target pairs showing negative correlations (P < 0.05) in IBD patients were filtered.

### LPS dose response of potential target genes of miR-374a-5p

Monocytes from HC were cultured (6 × 10^5^) in RPMI 1640 with 10% FBS (both from Sigma-Aldrich), and different concentrations (0.5, 2, 10, and 100 ng/ml) of LPS from *Escherichia coli* (Sigma-Aldrich) in ultra-low-attachment 24-well plates (Corning). After 24 h, the RNA was extracted using the RNA/DNA/Protein Purification Plus kit (Norgen Biotek Corp.). The expression of both miR-374a-5p and target genes was assessed by quantitative real-time PCR.

### Cell transfection

To select the optimal transfection conditions, 6 × 10^5^ HC monocytes were transfected with 50 or 100 nM of Cy3 Dye-Labeled Pre-miR Negative Control for 24 and 48 h using siPORT NeoFXTM transfection agent (Thermo Fisher Scientific). Cell viability was assessed by Annexin V FITC and PI staining. Flow cytometry analysis suggested that the mimic concentration of 100 nM for 48 h provided the highest ratio of transfection without compromising cell viability (Fig. S3 J).

HC and IBD monocytes were transfected for 48 h with 100 nM of miR-374a-5p miR mimic or negative scrambled control (miR Vana; Thermo Fisher Scientific). In HC, monocytes were activated with 2 ng/ml of LPS after 24 h of transfection. Finally, RNA and protein were extracted, and supernatants were stored at −80°C. miR-374a mimic transfection did not affect the growth and survival of the monocytes.

In parallel, HC monocytes were also transfected with 100 nM of miR-374a-5p inhibitor (miRVana; Thermo Fisher Scientific) or negative control (miRNA Inhibitor, negative control; miRVana; Thermo Fisher Scientific) for 48 h. RNA and protein were extracted, and supernatants were stored at −80°C.

### Quantitative real-time PCR

RNA samples were retrotranscribed using SuperScript VILO cDNA Synthesis Kit (Thermo Fisher Scientific) for gene expression analysis. TaqMan Expression Assays (Life Technologies) and LightCycler 480 System II (Roche Diagnostics) were used to assess the expression of miR-374a-5p and its potential target genes. The expression level of miR-374a-5p was normalized with RNU6B, whereas target genes were normalized with Cyclophilin B and β2-microglobulin. The 2^−ΔΔCt^ method was used to calculate relative changes in gene expression.

### Western blotting

Proteins were separated by SDS-PAGE using NuPAGE 4–12% Bis-Tris Gel (Invitrogen). Protein bands were then transferred onto a polyvinylidene difluoride membrane (Invitrogen). Absolute band intensities of the indicated proteins were captured and quantified with NIH ImageJ Lab software. Cyclophilin B was used as a loading control.

### Luminex assay for cytokine production in culture supernatants

Secreted levels of *TNFα*, *OSM*, *IL1A*, *IL6*, and *CCL18* were determined using a Magnetic Luminex Assay multiplex kit (R&D) following the manufacturer’s instructions. Analysis was performed on a Luminex MAGPIX Instrument (Luminex) with xPONENT software.

### Luciferase assays

The direct interaction between selected potential targets and miR-374a-5p was evaluated by using the LightSwitch Luciferase Assay reporter system (SwitchGear Genomics) following the manufacturer’s instructions. Briefly, 2.5 × 10^4^ HEK293 cells were seeded in 96-white-well plates (Thermo Fisher Scientific) 24 h before transfection. Then, cells were cotransfected with SwitchGear GoClone reporter constructs for each target and with 100 nM of either miR-374a-5p mimic or negative scrambled control for 24 h. Luciferase activity was detected by a luminometer. Negative controls included one empty vector and two vectors with no binding sites for miR-374a-5p (GAPDH and TNFα).

### Total RNA library prep, sequencing, and data analysis

Samples from LPS-stimulated HC monocytes pretransfected with miR-374a-5p mimic or negative scrambled control with an RNA integrity number >7 were used for RNA-seq library construction with the SMARTer Stranded Total RNA-Seq Kit Pico Input (Takara Bio), following the manufacturer’s instructions. Paired-end strategy 150-bp sequencing was performed on an Illumina HiSeq 4000 instrument. Quality control was performed using the FastQC program. Reads having a Phred score <24 were removed along with the adaptors, and the first three bases of both read 2 and read 1 using Trimgalore. Ribosomal RNA reads were removed using Bbsplit. Reads were mapped onto grch38 reference genome using HISAT2 and checked using QoRTs. Finally, a count table was generated, and the data were normalized using the R packages Rsubread and edgeR. Finally, GSEA was performed using all gene sets from the molecular signature database (C7 Immunologic gene sets), with an FDR < 0.05 considered significant.

### Migration assays

Cell migration was monitored using a 96-well ChemoTx Disposable Chemotaxis System (pore size, 5 µm; well capacity, 30 μl; cell site diameter, 3.2 mm; filter membranes, standard PCTE; Neuro Probe Inc.). Transfected monocytes or CD4^+^ T cells were incubated with 10 µM Calcein-AM (Thermo Fisher Scientific) for 30 min at 37°C and then washed in PBS. A 25-μl drop containing 2.5 × 10^4^ cells was placed in triplicate onto a 5-µm-pore membrane. For monocyte migration assays, 30 μl of CCL2 (10 nM) was placed in each well under the membrane, and for CD4 migration assays, 30 μl of supernatant from transfected monocytes were used as chemoattractant. After 1 h at 37°C, Calcein fluorescence was measured under the membrane at 517 nm in a CLARIOstar^Plus^ microplate reader (BMG Labtech). The percentage of migration was calculated by dividing by the fluorescence of the initial drop.

### CD4^+^ T cell and monocyte coculture

CD4^+^ T cells (1 × 10^5^) from HC and IBD patients were cultured in RPMI 1640 and 10% FBS in a flat-bottom anti-CD3–coated plate (10 µg/ml) with 6 × 10^4^ autologous monocytes transfected with miR-374a-5p mimic or scrambled as previously shown. After 24 h of coculture, the phenotype of CD4^+^ T cells was evaluated by flow cytometry.

### Flow cytometry analysis

Live/dead discrimination was performed using the LIVE/DEAD Fixable Aqua Dead Cell Stain Kit. The expression of activation, naive, and memory markers in CD4^+^ T cells was assessed by using a mix of CD4-eF450, CD25-BV780, HLA-DR-BV570, CD45RA-BV605, and CD62L-APC eF780 anti-human mAbs in a BD LSRFortessa cell analyzer (BD Biosciences). Data were analyzed using FlowJo software.

### Study approval

Ethical approval for this work was obtained from the East of England Cambridgeshire and Hertfordshire Ethics Committee (REC reference 08/H0306/21). All participants provided written informed consent.

### Online supplemental material

Fig. S1 depicts the preservation of gene expression modules across different datasets, the association of the Tan module and miR-374a-5p with anatomic location, and the relationship between TNFα expression and mir-374a-5p and other members of the inflammation module. Fig. S2 shows the expression of miR-374a-5p family members in different immune cell types and the results of transfection experiments with a mir374a-5p mimic and antagomiR on the expression levels of target genes. Fig. S3 shows the global transcriptional impact of miR-374a-5p overexpression, the functional consequences of coculturing CD4 T cells and monocytes from IBD patients transfected with miR374a-5p, and quality control data on the miRNA datasets described in this manuscript. Table S1 contains the clinical characteristics of patients, data on miRNAs DE between patients with IBD and HC, and information regarding known miR-374a-5p targets. Table S2 shows small RNA-seq analysis in monocytes (IBD vs. HC). Table S3 shows a description of targets of miR-374a-5p.

## Supplementary Material

Table S1 shows baseline characteristics of IBD patients.

Table S2 shows small RNA-seq analysis in monocytes (IBD vs. HC).

Table S3 shows a description of targets of miR-374a-5p.

## Data Availability

The microarray, miRNA-seq, and RNA-seq data described in this article have been deposited in ArrayExpress (accession nos. E-MTAB-3554, E-MTAB-2713, and E-MTAB-11565) or the European Genome-Phenome Archive under accession no. EGAS00001006157.

## References

[bib1] Agostini, M., and R.A. Knight. 2014. miR-34: from bench to bedside. Oncotarget. 5:872–881. 10.18632/oncotarget.1825 24657911 PMC4011589

[bib2] Alatab, S., S.G. Sepanlou, K. Ikuta, H. Vahedi, C. Bisignano, S. Safiri, A. Sadeghi, M.R. Nixon, A. Abdoli, H. Abolhassani, V. Alipour, . 2020. The global, regional, and national burden of inflammatory bowel disease in 195 countries and territories, 1990–2017: a systematic analysis for the global burden of disease study 2017. Lancet Gastroenterol. Hepatol. 5:17–30. 10.1016/s2468-1253(19)30333-4 31648971 PMC7026709

[bib3] Allocca, M., M. Jovani, G. Fiorino, S. Schreiber, and S. Danese. 2013. Anti-IL-6 treatment for inflammatory bowel diseases: next cytokine, next target. Curr. Drug Targets.. 14:1508–1521. 10.2174/13894501113146660224 24102406

[bib4] Anderson, C.A., G. Boucher, C.W. Lees, A. Franke, M. D’Amato, K.D. Taylor, J.C. Lee, P. Goyette, M. Imielinski, A. Latiano, . 2011. Meta-analysis identifies 29 additional ulcerative colitis risk loci, increasing the number of confirmed associations to 47. Nat. Genet.. 43:246–252. 10.1038/ng.764 21297633 PMC3084597

[bib5] Arias de la Rosa, I., C. Perez-Sanchez, P. Ruiz-Limon, A. Patiño-Trives, C. Torres-Granados, Y. Jimenez-Gomez, M.D.C. Abalos-Aguilera, I. Cecchi, R. Ortega, M.A. Caracuel, J. Calvo-Gutierrez, . 2020. Impaired microRNA processing in neutrophils from rheumatoid arthritis patients confers their pathogenic profile. Modulation by biological therapies. Haematologica. 105:2250–2261. 10.3324/haematol.2018.205047 33054050 PMC7556520

[bib6] Arijs, I., G. De Hertogh, K. Lemaire, R. Quintens, L. Van Lommel, K. Van Steen, P. Leemans, I. Cleynen, G. Van Assche, S. Vermeire, K. Geboes, . 2009a. Mucosal gene expression of antimicrobial peptides in inflammatory bowel disease before and after first infliximab treatment. PLoS One. 4:e7984. 10.1371/journal.pone.0007984 19956723 PMC2776509

[bib7] Arijs, I., G. De Hertogh, B. Lemmens, L. Van Lommel, M. De Bruyn, W. Vanhove, I. Cleynen, K. MacHiels, M. Ferrante, F. Schuit, G. Van Assche, . 2018. Effect of vedolizumab (anti-α4β7-integrin) therapy on histological healing and mucosal gene expression in patients with UC. Gut. 67:43–52. 10.1136/gutjnl-2016-312293 27802155

[bib8] Arijs, I., K. Li, G. Toedter, R. Quintens, L. Van Lommel, K. Van Steen, P. Leemans, G. De Hertogh, K. Lemaire, M. Ferrante, F. Schnitzler, . 2009b. Mucosal gene signatures to predict response to infliximab in patients with ulcerative colitis. Gut. 58:1612–1619. 10.1136/gut.2009.178665 19700435

[bib9] Azam, M.A., and N.S. Tripuraneni. 2014. Selective Phosphodiesterase 4B Inhibitors: A Review. Sci. Pharm.. 82:453–481. 10.3797/scipharm.1404-08 25853062 PMC4318138

[bib10] Baillie, J.K., E. Arner, C. Daub, M. De Hoon, M. Itoh, H. Kawaji, T. Lassmann, P. Carninci, A.R.R. Forrest, Y. Hayashizaki; FANTOM Consortium, . 2017. Analysis of the human monocyte-derived macrophage transcriptome and response to lipopolysaccharide provides new insights into genetic aetiology of inflammatory bowel disease. PLoS Genet. 13:e1006641. 10.1371/journal.pgen.1006641 28263993 PMC5358891

[bib11] Baltimore, D., M.P. Boldin, R.M. O’Connell, D.S. Rao, and K.D. Taganov. 2008. MicroRNAs: new regulators of immune cell development and function. Nat. Immunol. 9:839–845. 10.1038/ni.f.209 18645592

[bib12] Bao, S., J. Zheng, N. Li, C. Huang, M. Chen, Q. Cheng, K. Yu, S. Chen, M. Zhu, and G. Shi. 2017. Serum MicroRNA levels as a noninvasive diagnostic biomarker for the early diagnosis of hepatitis B virus-related liver fibrosis. Gut Liver. 11:860–869. 10.5009/gnl16560 28750488 PMC5669603

[bib13] Barrett, J.C., S. Hansoul, D.L. Nicolae, J.H. Cho, R.H. Duerr, J.D. Rioux, S.R. Brant, M.S. Silverberg, K.D. Taylor, M.M. Barmada, . 2008. Genome-wide association defines more than 30 distinct susceptibility loci for Crohn’s disease. Nat. Genet.. 40:955–962. 10.1038/ng.175 18587394 PMC2574810

[bib14] Biasci, D., J.C. Lee, N.M. Noor, D.R. Pombal, M. Hou, N. Lewis, T. Ahmad, A. Hart, M. Parkes, E.F. McKinney, P.A. Lyons, . 2019. A blood-based prognostic biomarker in IBD. Gut. 68:1386–1395. 10.1136/gutjnl-2019-318343 31030191 PMC6691955

[bib15] Bourges, C., A.F. Groff, O.S. Burren, C. Gerhardinger, K. Mattioli, A. Hutchinson, T. Hu, T. Anand, M.W. Epping, C. Wallace, K.G. Smith, . 2020. Resolving mechanisms of immune-mediated disease in primary CD 4 T cells. EMBO Mol. Med. 12:e12112. 10.15252/emmm.202012112 32239644 PMC7207160

[bib16] Chen, X., C. Jia, C. Jia, X. Jin, and X. Gu. 2018. MicroRNA-374a inhibits aggressive tumor biological behavior in bladder carcinoma by suppressing Wnt/β-catenin signaling. Cell. Physiol. Biochem. 48:815–826. 10.1159/000491911 30032142

[bib17] Chen, Y., J. Jiang, M. Zhao, X. Luo, Z. Liang, Y. Zhen, Q. Fu, X. Deng, X. Lin, L. Li, R. Luo, . 2016. microRNA-374a suppresses colon cancer progression by directly reducing CCND1 to inactivate the PI3K/AKT pathway. Oncotarget. 7:41306–41319. 10.18632/oncotarget.9320 27191497 PMC5173061

[bib18] Chen, Z., Y. Hu, R. Lu, M. Ge, and L. Zhang. 2020. MicroRNA-374a-5p inhibits neuroinflammation in neonatal hypoxic-ischemic encephalopathy via regulating NLRP3 inflammasome targeted Smad6. Life Sci. 252:117664. 10.1016/j.lfs.2020.117664 32304765

[bib19] Cretney, E., P.S. Leung, S. Trezise, D.M. Newman, L.C. Rankin, C.E. Teh, T.L. Putoczki, D.H. Gray, G.T. Belz, L.A. Mielke, . 2018. Characterization of Blimp-1 function in effector regulatory T cells. J. Autoimmun.. 91:73–82. 10.1016/j.jaut.2018.04.003 29724515

[bib20] Doumatey, A.P., W.J. He, A. Gaye, L. Lei, J. Zhou, G.H. Gibbons, A. Adeyemo, and C.N. Rotimi. 2018. Circulating MiR-374a-5p is a potential modulator of the inflammatory process in obesity. Sci. Rep. 8:7680. 10.1038/s41598-018-26065-5 29769661 PMC5955981

[bib21] Dower, K., D.K. Ellis, K. Saraf, S.A. Jelinsky, and L.-L. Lin. 2008. Innate immune responses to TREM-1 activation: overlap, divergence, and positive and negative cross-talk with bacterial lipopolysaccharide. J. Immunol. 180:3520–3534. 10.4049/jimmunol.180.5.3520 18292579

[bib22] Ellinghaus, D., L. Jostins, S.L. Spain, A. Cortes, J. Bethune, B. Han, Y.R. Park, S. Raychaudhuri, J.G. Pouget, M. Hübenthal, . 2016. Analysis of five chronic inflammatory diseases identifies 27 new associations and highlights disease-specific patterns at shared loci.. Nat. Genet.. 48:510–518. 10.1038/ng.3528 26974007 PMC4848113

[bib23] Estep, M., D. Armistead, N. Hossain, H. Elarainy, Z. Goodman, A. Baranova, V. Chandhoke, and Z.M. Younossi. 2010. Differential expression of miRNAs in the visceral adipose tissue of patients with non-alcoholic fatty liver disease. Aliment. Pharmacol. Ther. 32:487–497. 10.1111/j.1365-2036.2010.04366.x 20497147

[bib24] Franke, A., D.P.B. McGovern, J.C. Barrett, K. Wang, G.L. Radford-Smith, T. Ahmad, C.W. Lees, T. Balschun, J. Lee, R. Roberts, . 2010. Genome-wide meta-analysis increases to 71 the number of confirmed Crohn's disease susceptibility loci. Nat. Genet.. 42:1118–1125. 10.1038/ng.717 21102463 PMC3299551

[bib25] Gong, W., S. Qie, P. Huang, and J. Xi. 2018. Protective effect of miR-374a on chemical hypoxia-induced damage of PC12 cells in vitro via the GADD45α/JNK signaling pathway. Neurochem. Res. 43:581–590. 10.1007/s11064-017-2452-0 29247275

[bib26] Goodman, W.A., S. Omenetti, D. Date, L. Di Martino, C. De Salvo, G.-D. Kim, S. Chowdhry, G. Bamias, F. Cominelli, T.T. Pizarro, and GH Mahabeleshwar. 2016. KLF6 contributes to myeloid cell plasticity in the pathogenesis of intestinal inflammation. Mucosal Immunol. 9:1250–1262. 10.1038/mi.2016.1 26838049 PMC4972715

[bib27] Häsler, R., R. Sheibani-Tezerji, A. Sinha, M. Barann, A. Rehman, D. Esser, K. Aden, C. Knecht, B. Brandt, S. Nikolaus, S. Schäuble, . 2017. Uncoupling of mucosal gene regulation, mRNA splicing and adherent microbiota signatures in inflammatory bowel disease. Gut. 66:2087–2097. 10.1136/gutjnl-2016-311651 27694142 PMC5749366

[bib28] Heras-Palou, C. 2019. Patisiran’s path to approval as an RNA therapy. Nature. 574:S7. 10.1038/d41586-019-03070-w 31619801

[bib29] Hildebrand, D.G., E. Alexander, S. Hörber, S. Lehle, K. Obermayer, N.-A. Münck, O. Rothfuss, J.-S. Frick, M. Morimatsu, I. Schmitz, . 2013. J. Immunol.. 190:4812–4820. 10.4049/jimmunol.1300089 23547114

[bib30] Hong, S.N., J.G. Joung, J.S. Bae, C.S. Lee, J.S. Koo, S.J. Park, J.P. Im, Y.S. Kim, J.W. Kim, W.Y. Park, and Y.H. Kim. 2017. RNA-seq reveals transcriptomic differences in inflamed and noninflamed intestinal mucosa of crohn’s disease patients compared with normal mucosa of healthy controls. Inflamm. Bowel Dis. 23:1098–1108. 10.1097/MIB.0000000000001066 28613228

[bib31] Huang, Z.Q., W. Xu, J.L. Wu, X. Lu, and X.M. Chen. 2019. MicroRNA-374a protects against myocardial ischemia-reperfusion injury in mice by targeting the MAPK6 pathway. Life Sci. 232:116619. 10.1016/j.lfs.2019.116619 31265855

[bib32] Isenberg, D.A., A. Rahman, E. Allen, V. Farewell, M. Akil, I.N. Bruce, D. D’Cruz, B. Griffiths, M. Khamashta, P. Maddison, N. McHugh, . 2005. BILAG 2004. Development and initial validation of an updated version of the British Isles Lupus Assessment Group’s disease activity index for patients with systemic lupus erythematosus. Rheumatology. 44:902–906. 10.1093/rheumatology/keh624 15814577

[bib33] Jones, M.F., T. Hara, P. Francis, X.L. Li, S. Bilke, Y. Zhu, M. Pineda, M. Subramanian, W.F. Bodmer, and A. Lal. 2015. The CDX1-microRNA-215 axis regulates colorectal cancer stem cell differentiation. Proc. Natl. Acad. Sci. USA. 112:E1550–E1558. 10.1073/pnas.1503370112 25775580 PMC4386393

[bib34] Jostins, L., S. Ripke, R.K. Weersma, R.H. Duerr, D.P. McGovern, K.Y. Hui, J.C. Lee, L.P. Schumm, Y. Sharma, C.A. Anderson, . 2012. Host-microbe interactions have shaped the genetic architecture of inflammatory bowel disease. Nature. 491:119–124. 10.1038/nature11582 23128233 PMC3491803

[bib35] Kalla, R., N.T. Ventham, N.A. Kennedy, J.F. Quintana, E.R. Nimmo, A.H. Buck, and J. Satsangi. 2015. MicroRNAs: new players in IBD. Gut. 64:504–517. 10.1136/gutjnl-2014-307891 25475103 PMC4345829

[bib36] Kashmiry, A., R. Tate, G. Rotondo, J. Davidson, and D. Rotondo. 2018. The prostaglandin EP4 receptor is a master regulator of the expression of PGE 2 receptors following inflammatory activation in human monocytic cells. Biochim. Biophys. Acta Mol. Cell Biol. Lipids.. 1863:1297–1304. 10.1016/j.bbalip.2018.07.003 30053598

[bib37] Keith, B.P., J.B. Barrow, T. Toyonaga, N. Kazgan, M.H. O’Connor, N.D. Shah, M.S. Schaner, E.A. Wolber, O.K. Trad, G.R. Gipson, W.A. Pitman, . 2018. Colonic epithelial miR-31 associates with the development of Crohn’s phenotypes. JCI Insight. 3:e122788. 10.1172/jci.insight.122788 PMC6237459 30282822

[bib38] Langfelder, P., and S. Horvath. 2008. WGCNA: an R package for weighted correlation network analysis. BMC Bioinformatics. 9:559. 10.1186/1471-2105-9-559 19114008 PMC2631488

[bib39] Langfelder, P., R. Luo, M.C. Oldham, and S. Horvath. 2011. Is my network module preserved and reproducible? PLoS Comput. Biol. 7:e1001057. 10.1371/journal.pcbi.1001057 21283776 PMC3024255

[bib40] Li, Z., and T.M. Rana. 2014. Therapeutic targeting of microRNAs: current status and future challenges. Nat. Rev. Drug Discov. 13:622–638. 10.1038/nrd4359 25011539

[bib41] Libioulle, C., E. Louis, S. Hansoul, C. Sandor, F. Farnir, D. Franchimont, S. Vermeire, O. Dewit, M. de Vos, A. Dixon, . 2007. Novel Crohn disease locus identified by genome-wide association maps to a gene desert on 5p13.1 and modulates expression of PTGER4. PLoS Genet. 3:e58. 10.1371/journal.pgen.0030058 17447842 PMC1853118

[bib42] Looney, A.M., B.H. Walsh, G. Moloney, S. Grenham, A. Fagan, G.W. O’Keeffe, G. Clarke, J.F. Cryan, T.G. Dinan, G.B. Boylan, and D.M. Murray. 2015. Downregulation of umbilical cord blood levels of miR-374a in neonatal hypoxic ischemic encephalopathy. J. Pediatr. 167:269–273.e2. 10.1016/j.jpeds.2015.04.060 26001314

[bib43] Liu, J.Z., S. van Sommeren, H. Huang, S.C. Ng, R. Alberts, A. Takahashi, S. Ripke, J.C. Lee, L. Jostins, T. Shah, . 2015. Association analyses identify 38 susceptibility loci for inflammatory bowel disease and highlight shared genetic risk across populations. Nat. Genet.. 47:979–986. 10.1038/ng.3359 26192919 PMC4881818

[bib44] Luo, X., L. Zhang, M. Li, W. Zhang, X. Leng, F. Zhang, Y. Zhao, and X. Zeng. 2013. The role of miR-125b in T lymphocytes in the pathogenesis of systemic Lupus erythematosus. Clin. Exp. Rheumatol. 31:263–271. 23305626

[bib45] Lyons, P.A., M. Koukoulaki, A. Hatton, K. Doggett, H.B. Woffendin, A.N. Chaudhry, and K.G.C. Smith. 2007. Microarray analysis of human leucocyte subsets: the advantages of positive selection and rapid purification. BMC Genomics. 8:64. 10.1186/1471-2164-8-64 17338817 PMC1828063

[bib46] Lyons, P.A., E.F. McKinney, T.F. Rayner, A. Hatton, H.B. Woffendin, M. Koukoulaki, T.C. Freeman, D.R.W. Jayne, A.N. Chaudhry, and K.G.C. Smith. 2010. Novel expression signatures identified by transcriptional analysis of separated leucocyte subsets in systemic lupus erythematosus and vasculitis. Ann. Rheum. Dis. 69:1208–1213. 10.1136/ard.2009.108043 19815495 PMC2935323

[bib47] Malik, A., and T.-D. Kanneganti. 2018. Function and regulation of IL-1α in inflammatory diseases and cancer. Immunol. Rev.. 281:124–137. 10.1111/imr.12615 29247991 PMC5739076

[bib48] Matkovich, S.J., Y. Hu, W.H. Eschenbacher, L.E. Dorn, and G.W. Dorn. 2012. Direct and indirect involvement of MicroRNA-499 in clinical and experimental cardiomyopathy. Circ. Res. 111:521–531. 10.1161/CIRCRESAHA.112.265736 22752967 PMC3429338

[bib49] Na, Y.R., M. Stakenborg, S.H. Seok, and G. Matteoli. 2019. Macrophages in intestinal inflammation and resolution: a potential therapeutic target in IBD. Nat. Rev. Gastroenterol. Hepatol. 16:531–543. 10.1038/s41575-019-0172-4 31312042

[bib50] Neurath, M.F. 2019. Targeting immune cell circuits and trafficking in inflammatory bowel disease. Nat. Immunol. 20:970–979. 10.1038/s41590-019-0415-0 31235952

[bib89] Okamoto, K., Y. Iwai, M. Oh-Hora, M. Yamamoto, T. Morio, K. Aoki, K. Ohya, A.M. Jetten, S. Akira, T. Muta, . 2010. IkappaBzeta regulates T(H)17 development by cooperating with ROR nuclear receptors. Nature. 464:1381–1385. 10.1038/nature08922 20383124

[bib51] Patial, S., and P.J. Blackshear. 2016. Tristetraprolin as a Therapeutic Target in Inflammatory Disease. Trends Pharmacol. Sci.. 37:811–821. 10.1016/j.tips.2016.07.002 27503556 PMC5030171

[bib52] Pérez-Sánchez, C., M.A. Aguirre, P. Ruiz-Limón, N. Barbarroja, Y. Jiménez-Gómez, I.A. De La Rosa, A. Rodriguez-Ariza, E. Collantes-Estévez, P. Segui, F. Velasco, M.J. Cuadrado, . 2016. Atherothrombosis-associated microRNAs in antiphospholipid syndrome and systemic lupus erythematosus patients. Sci. Rep. 6:31375. 10.1038/srep31375 27502756 PMC4977549

[bib53] Pérez-Sánchez, C., I. Arias-de la Rosa, M.Á. Aguirre, M. Luque-Tévar, P. Ruiz-Limón, N. Barbarroja, Y. Jiménez-Gómez, M.C. Ábalos-Aguilera, E. Collantes-Estévez, P. Segui, F. Velasco, . 2018. Circulating microRNAs as biomarkers of disease and typification of the atherothrombotic status in antiphospholipid syndrome. Haematologica. 103:908–918. 10.3324/haematol.2017.184416 29545345 PMC5927979

[bib54] Podolsky, D.K. 2002. Inflammatory bowel disease. N. Engl. J. Med. 347:417–429. 10.1056/nejmra020831 12167685

[bib55] Prajzlerová, K., K. Grobelná, M. Hušáková, Š. Forejtová, A. Jüngel, S. Gay, J. Vencovský, K. Pavelka, L. Šenolt, and M. Filková. 2017. Association between circulating miRNAs and spinal involvement in patients with axial spondyloarthritis. PLoS One. 12:e0185323. 10.1371/journal.pone.0185323 28938006 PMC5609864

[bib56] Rawat, M., M. Nighot, R. Al-Sadi, Y. Gupta, D. Viszwapriya, G. Yochum, W. Koltun, and T.Y. Ma. 2020. IL1B increases intestinal tight junction permeability by up-regulation of MIR200C-3p, which degrades occludin mRNA. Gastroenterology. 159:1375–1389. 10.1053/j.gastro.2020.06.038 32569770 PMC11752806

[bib57] Rupaimoole, R., H.-D. Han, G. Lopez-Berestein, and A.K. Sood. 2011. MicroRNA therapeutics: principles, expectations, and challenges. Chin. J. Cancer. 30:368–370. 10.5732/cjc.011.10186 21627858 PMC4013410

[bib88] Ross, E.A., A.J. Naylor, J.D. O’Neil, T. Crowley, M.L. Ridley, J. Crowe, T. Smallie, T.J. Tang, J.D. Turner, L.V. Norling, . 2017. Treatment of inflammatory arthritis via targeting of tristetraprolin, a master regulator of pro-inflammatory gene expression. Ann. Rheum. Dis. 76:612–619. 10.1136/annrheumdis-2016-209424 27597652 PMC5446007

[bib58] Rupaimoole, R., and F.J. Slack. 2017. MicroRNA therapeutics: towards a new era for the management of cancer and other diseases. Nat. Rev. Drug Discov. 16:203–222. 10.1038/nrd.2016.246 28209991

[bib59] Salehi, S., R. Bankoti, L. Benevides, J. Willen, M. Couse, J.S. Silva, D. Dhall, E. Meffre, S. Targan, and G.A. Martins. 2012. B lymphocyte-induced maturation protein-1 contributes to intestinal mucosa homeostasis by limiting the number of IL-17-producing CD4+ T cells. J. Immunol.. 189:5682–5693. 10.4049/jimmunol.1201966 23162130 PMC3529138

[bib60] Schaefer, J.S. 2016. MicroRNAs: how many in inflammatory bowel disease? Curr. Opin. Gastroenterol. 32:258–266. 10.1097/mog.0000000000000284 27138057 PMC5659191

[bib61] Schaefer, J.S., T. Attumi, A.R. Opekun, B. Abraham, J. Hou, H. Shelby, D.Y. Graham, C. Streckfus, and J.R. Klein. 2015. MicroRNA signatures differentiate Crohn’s disease from ulcerative colitis. BMC Immunol. 16:5. 10.1186/S12865-015-0069-0 25886994 PMC4335694

[bib62] Schrier, S.B., A.S. Hill, D. Plana, and D.A. Lauffenburger. 2016. Synergistic communication between CD4+ T cells and monocytes impacts the cytokine environment. Sci. Rep. 6:34942. 10.1038/srep34942 27721433 PMC5056362

[bib87] Schutyser, E., A. Richmond, and J. Van Damme. 2005. Involvement of CC chemokine ligand 18 (CCL18) in normal and pathological processes. J. Leukoc. Biol. 78:14–26. 10.1189/jlb.1204712 15784687 PMC2665283

[bib63] Seumois, G., J. Zapardiel-Gonzalo, B. White, D. Singh, V. Schulten, M. Dillon, D. Hinz, D.H. Broide, A. Sette, B. Peters, and P Vijayanand. 2016. Transcriptional Profiling of Th2 Cells Identifies Pathogenic Features Associated with Asthma. J. Immunol.. 197:655–664. 10.4049/jimmunol.1600397 27271570 PMC4936908

[bib64] Shahab, S.W., L. V Matyunina, C.G. Hill, L. Wang, R. Mezencev, L. DeEtte Walker, and J.F. McDonald. 2012. The effects of MicroRNA transfections on global patterns of gene expression in ovarian cancer cells are functionally coordinated. BMC Med. Genomics. 5:33. 10.1186/1755-8794-5-33 22853714 PMC3481362

[bib65] Shaw, K.K., L.C. Rausch-Derra, and L. Rhodes. 2016. Grapiprant: an EP4 prostaglandin receptor antagonist and novel therapy for pain and inflammation. Vet. Med. Sci.. 2:3–9. 10.1002/vms3.13 29067176 PMC5645826

[bib66] Shi, F.L., and L.X. Ren. 2020. Up-regulated miR-374a-3p relieves lipopolysaccharides induced injury in CHON-001 cells via regulating wingless-type MMTV integration site family member 5B. Mol. Cell. Probes. 51:101541. 10.1016/j.mcp.2020.101541 32092330

[bib67] Silverberg, M.S., J. Satsangi, T. Ahmad, I.D.R. Arnott, C.N. Bernstein, S.R. Brant, R. Caprilli, J.F. Colombel, C. Gasche, K. Geboes, D.P. Jewell, . 2005. Toward an integrated clinical, molecular and serological classification of inflammatory bowel disease: report of a working party of the 2005 Montreal World Congress of Gastroenterology. Can. J. Gastroenterol. 19:5A–36A. 10.1155/2005/269076 16151544

[bib68] Spadaccini, M., S. D’Alessio, L. Peyrin-Biroulet, and S. Danese. 2017. PDE4 Inhibition and Inflammatory Bowel Disease: A Novel Therapeutic Avenue. Int. J. Mol. Sci.. 18:1276:E1276. 10.3390/ijms18061276 PMC5486098 28617319

[bib69] Stępień, E., M. Durak-Kozica, A. Kamińska, M. Targosz-Korecka, M. Libera, G. Tylko, A. Opalińska, M. Kapusta, B. Solnica, A. Georgescu, . 2018. Circulating ectosomes: determination of angiogenic microRNAs in type 2 diabetes. Theranostics. 8:3874–3890. 10.7150/thno.23334 30083267 PMC6071541

[bib70] Stone, J.H., G.S. Hoffman, P.A. Merkel, Y.I. Min, M.L. Uhlfelder, D.B. Hellmann, U. Specks, N.B. Allen, J.C. Davis, R.F. Spiera, L.H. Calabrese, ; for the International Network for the Study of the Systemic Vasculitides (INSSYS). 2001. A disease-specific activity index for Wegener’s granulomatosis: modification of the Birmingham vasculitis activity score. International network for the study of the systemic vasculitides (INSSYS). Arthritis Rheum. 44:912–920. 10.1002/1529-0131(200104)44:4<912::aid-anr148>3.0.co;2-5 11318006

[bib71] Tan, E.M., A.S. Cohen, J.F. Fries, A.T. Masi, D.J. Mcshane, N.F. Rothfield, J.G. Schaller, N. Talal, and R.J. Winchester. 1982. The 1982 revised criteria for the classification of systemic lupus erythematosus. Arthritis Rheum. 25:1271–1277. 10.1002/art.1780251101 7138600

[bib72] Thomas, M., J. Lieberman, and A. Lal. 2010. Desperately seeking microRNA targets. Nat. Struct. Mol. Biol. 17:1169–1174. 10.1038/nsmb.1921 20924405

[bib73] Thomas, H.. 2017. IBD: Oncostatin M promotes inflammation in IBD. Nat. Rev. Gastroenterol. Hepatol.. 14:261. 10.1038/nrgastro.2017.47 28400625

[bib74] Van Den Hoogen, L.L., M. Rossato, A.P. Lopes, A. Pandit, C.P.J. Bekker, R.D.E. Fritsch-Stork, J.A.G. Van Roon, and T.R.D.J. Radstake. 2018. MicroRNA downregulation in plasmacytoid dendritic cells in interferon-positive systemic lupus erythematosus and antiphospholipid syndrome. Rheumatol. (Oxford). 57:1669–1674. 10.1093/rheumatology/key159 29873766

[bib75] Van Der Goten, J., W. Vanhove, K. Lemaire, L. Van Lommel, K. Machiels, W.J. Wollants, V. De Preter, G. De Hertogh, M. Ferrante, G. Van Assche, P. Rutgeerts, . 2014. Integrated miRNA and mRNA expression profiling in inflamed colon of patients with ulcerative colitis. PLoS One. 9:e116117. 10.1371/journal.pone.0116117 25546151 PMC4278881

[bib76] Vereecke, L., M. Sze, C. Mc Guire, B. Rogiers, Y. Chu, M. Schmidt-Supprian, M. Pasparakis, R. Beyaert, and G. van Loo. 2010. Enterocyte-specific A20 deficiency sensitizes to tumor necrosis factor-induced toxicity and experimental colitis. J. Exp. Med.. 207:1513–1523. 10.1084/jem.20092474 20530205 PMC2901067

[bib77] Verstockt, S., G. De Hertogh, J. Van Der Goten, B. Verstockt, M. Vancamelbeke, K. Machiels, L. Van Lommel, F. Schuit, G. Van Assche, P. Rutgeerts, M. Ferrante, . 2019. Gene and mirna regulatory networks during different stages of Crohn’s disease. J. Crohns Colitis. 13:916–930. 10.1093/ecco-jcc/jjz007 30657881

[bib78] Waldner, M.J., and M.F. Neurath. 2014. Master regulator of intestinal disease: IL-6 in chronic inflammation and cancer development. Semin. Immunol.. 26:75–79. 10.1016/j.smim.2013.12.003 24447345

[bib79] Walsh, A.J., R. V. Bryant, and S.P.L. Travis. 2016. Current best practice for disease activity assessment in IBD. Nat. Rev. Gastroenterol. Hepatol. 13:567–579. 10.1038/nrgastro.2016.128 27580684

[bib80] Wang-Renault, S.F., S. Boudaoud, G. Nocturne, E. Roche, N. Sigrist, C. Daviaud, A.B. Tinggaard, V. Renault, J.F. Deleuze, X. Mariette, and J. Tost. 2018. Deregulation of microRNA expression in purified T and B lymphocytes from patients with primary Sjögren’s syndrome. Ann. Rheum. Dis. 77:133–140. 10.1136/annrheumdis-2017-211417 28916716 PMC5754740

[bib81] West, N.R., A.N. Hegazy, B.M.J. Owens, S.J. Bullers, B. Linggi, S. Buonocore, M. Coccia, D. Görtz, S. This, K. Stockenhuber, J. Pott, . 2017. Oncostatin M drives intestinal inflammation and predicts response to tumor necrosis factor–neutralizing therapy in patients with inflammatory bowel disease. Nat. Med. 23:579–589. 10.1038/nm.4307 28368383 PMC5420447

[bib82] Williams, A.E., H. Larner-Svensson, M.M. Perry, G.A. Campbell, S.E. Herrick, I.M. Adcock, J.S. Erjefalt, K.F. Chung, and M.A. Lindsay. 2009. MicroRNA expression profiling in mild asthmatic human airways and effect of corticosteroid therapy. PLoS One. 4:e5889. 10.1371/journal.pone.0005889 19521514 PMC2690402

[bib83] Wu, H., Y. Liu, X.O. Shu, and Q. Cai. 2016. MiR-374a suppresses lung adenocarcinoma cell proliferation and invasion by targeting TGFA gene expression. Carcinogenesis. 37:567–575. 10.1093/carcin/bgw038 27207663 PMC4876989

[bib84] Yang, Z., Z. Guo, J. Dong, S. Sheng, Y. Wang, L. Yu, H. Wang, and L. Tang. 2018. miR-374a regulates inflammatory response in diabetic nephropathy by targeting MCP-1 expression. Front. Pharmacol. 9:900. 10.3389/fphar.2018.00900 30147653 PMC6095963

[bib85] Zhang, M., K. Sun, Y. Wu, Y. Yang, P. Tso, and Z. Wu. 2017. Interactions between Intestinal microbiota and host immune response in inflammatory bowel disease. Front. Immunol. 8:942. 10.3389/fimmu.2017.00942 28855901 PMC5558048

[bib86] Zhang, S., X. Ouyang, X. Jiang, D. Gu, Y. Lin, S.K. Kong, and W. Xie. 2015. Dysregulated serum microRNA expression profile and potential biomarkers in hepatitis C virus-infected patients. Int. J. Med. Sci. 12:590–598. 10.7150/ijms.11525 26283876 PMC4532963

