# Supplementary material for: miR-374a-5p regulates inflammatory genes and monocyte function in patients with inflammatory bowel disease
Source: J Exp Med. 2022 Apr 1;219(5):e20211366. doi: 10.1084/jem.20211366 (PMC8980842; doi:10.1084/jem.20211366)
Supplement: Table S1 — shows baseline characteristics of IBD patients. [file JEM_20211366_TableS1.docx]

**Table S1.** Baseline characteristics of IBD patients

|  | HC (20) | CD (21) | UC (25) | P | P | P |
| --- | --- | --- | --- | --- | --- | --- |
|  |  |  |  | (HC vs. CD) | (HC vs. UC) | (CD vs. UC) |
| Age (yr) | 41.6 (27–53.5) | 35.67 (23.5–45.5) | 44 (32.5–51.5) | 0.23 | 0.53 | 0.08 |
| Gender (F) | 17 (85%) | 14 (66%) | 15 (60%) | 0.27 | 0.1 | 0.76 |
| HBSI |  | 10.9 (8–12) | - |  |  |  |
| SSCI |  | - | 8.96 (8–11) |  |  |  |
| CRP (mg/liter) |  | 39.14 (10–57) | 16.27 (4–24) |  |  | 0.06 |
| ESR (mm/h) |  | 29.41 (14.5–42.5) | 22 (12–30) |  |  | 0.38 |
| Hemoglobin (g/dl) |  | 12.49 (11.4–14.1) | 13.56 (12.6–14.4) |  |  | 0.12 |
| Albumin (g/liter) |  | 35.13 | 39.69 |  |  |  |
| ASCA seropositive |  | 10 (50%) | - |  |  |  |
| Endoscopy (%moderate/severe) |  | 70.6 | 53.85 |  |  | 0.45 |
| Disease distribution |  |  |  |  |  |  |
| CD-L1 (ileal) |  | 5 | - |  |  |  |
| CD-L2 (colonic) |  | 4 | - |  |  |  |
| CD-L3 (ileocolonic) |  | 11 | - |  |  |  |
| CD-L4 (upper GI) |  | 1 | - |  |  |  |
| Perianal |  | 3 | - |  |  |  |
| UC-E1 (proctitis) |  | - | 6 |  |  |  |
| UC-E2 (left-sided) |  | - | 9 |  |  |  |
| UC-E3 (extensive) |  | - | 10 |  |  |  |

Data shown in parentheses indicate the interquartile range or percentages when indicated. Statistical significance was assessed using a Mann-Whitney test (two-tailed) or a Fisher’s exact test for dichotomous variables (gender and endoscopy). Disease distribution was classified according to Montreal Classification (Silverberg et al., 2005). Normal range: CRP, 0–6 mg/liter; ESR, 1–10 mm/h; hemoglobin, 13.5–17.2 g/dl; Albumin, 35–50 g/liter. HBSI, Harvey-Bradshaw severity index; SSCI, simple clinical colitis activity index; GI, gastrointestinal.

Reference

Silverberg, M.S., J. Satsangi, T. Ahmad, I.D.R. Arnott, C.N. Bernstein, S.R. Brant, R. Caprilli, J.F. Colombel, C. Gasche, K. Geboes, D.P. Jewell, et al. 2005. Toward an integrated clinical, molecular and serological classification of inflammatory bowel disease: report of a working party of the 2005 Montreal World Congress of Gastroenterology. *Can. J. Gastroenterol.* 19:5A–36A. 10.1155/2005/269076
