# Supplementary material for: miR-374a-5p regulates inflammatory genes and monocyte function in patients with inflammatory bowel disease
Source: J Exp Med. 2022 Apr 1;219(5):e20211366. doi: 10.1084/jem.20211366 (PMC8980842; doi:10.1084/jem.20211366)
Supplement: Table S2 — shows small RNA-seq analysis in monocytes (IBD vs. HC). [file JEM_20211366_TableS2.docx]

**Table S2.** Small RNA-seq analysis in monocytes

*MiRs downregulated (IBD vs. HC)*

| **miR** | **FDR**  **<0.05** | **FC log_2_** | **CPM**  **log_10_** | **miR** | **FDR**  **>0.05** | **FC**  **log_2_** | **CPM**  **log_10_** |
| --- | --- | --- | --- | --- | --- | --- | --- |
| **hsa-miR-24-2-5p** | **1.2E-09** | **-0.7** | **2.16** | hsa-miR-130b-5p | 5.1E-02 | -0.3 | 2.69 |
| **hsa-miR-19b-3p** | **1.2E-06** | **-1.0** | **2.85** | hsa-miR-3607-3p | 5.5E-02 | -0.6 | 1.34 |
| **hsa-miR-19a-3p** | **1.8E-06** | **-1.1** | **2.35** | hsa-miR-2355-3p | 5.6E-02 | -0.4 | 0.64 |
| **hsa-miR-374a-5p** | **4.8E-06** | **-1.0** | **2.11** | hsa-miR-199b-5p | 5.8E-02 | -0.3 | 2.66 |
| **hsa-miR-335-5p** | **5.1E-06** | **-1.1** | **2.09** | hsa-miR-4662a-5p | 6.1E-02 | -0.6 | 1.17 |
| **hsa-miR-590-3p** | **8.3E-06** | **-1.4** | **1.83** | hsa-miR-24-3p | 7.1E-02 | -0.4 | 2.87 |
| **hsa-miR-29b-3p** | **1.8E-05** | **-0.8** | **1.40** | hsa-miR-450a-5p | 7.1E-02 | -0.4 | 1.75 |
| **hsa-miR-7-5p** | **2.4E-05** | **-1.1** | **0.31** | hsa-miR-140-5p | 7.5E-02 | -0.3 | 2.30 |
| **hsa-miR-29c-3p** | **4.2E-05** | **-0.7** | **2.27** | hsa-miR-148b-3p | 9.4E-02 | -0.3 | 3.36 |
| **hsa-miR-548e-3p** | **4.9E-05** | **-0.8** | **1.95** | hsa-miR-660-3p | 9.7E-02 | -0.3 | 0.65 |
| **hsa-miR-450b-5p** | **1.4E-04** | **-1.0** | **2.08** | hsa-miR-335-3p | 1.0E-01 | -0.5 | 1.09 |
| **hsa-miR-429** | **1.9E-04** | **-0.9** | **0.74** | hsa-miR-18a-3p | 1.1E-01 | -0.2 | 1.83 |
| **hsa-miR-7-1-3p** | **2.1E-04** | **-0.7** | **1.32** | hsa-miR-6513-3p | 1.1E-01 | -0.3 | 0.56 |
| **hsa-miR-33a-5p** | **2.6E-04** | **-0.9** | **1.25** | hsa-miR-101-5p | 1.1E-01 | -0.4 | 1.47 |
| **hsa-miR-1277-5p** | **3.0E-04** | **-1.1** | **1.18** | hsa-miR-576-5p | 1.1E-01 | -0.3 | 1.72 |
| **hsa-miR-23a-5p** | **3.9E-04** | **-2.3** | **0.83** | hsa-miR-31-5p | 1.2E-01 | -0.6 | 0.80 |
| **hsa-miR-652-5p** | **4.1E-04** | **-0.8** | **0.94** | hsa-miR-4286 | 1.3E-01 | -0.4 | 2.36 |
| **hsa-miR-126-3p** | **4.1E-04** | **-0.6** | **1.87** | hsa-miR-2355-5p | 1.3E-01 | -0.3 | 1.10 |
| **hsa-miR-17-3p** | **4.2E-04** | **-0.4** | **1.96** | hsa-miR-107 | 1.3E-01 | -0.1 | 3.04 |
| **hsa-miR-374a-3p** | **4.9E-04** | **-0.8** | **2.42** | hsa-miR-616-5p | 1.3E-01 | -0.3 | 1.23 |
| **hsa-miR-1537-3p** | **5.3E-04** | **-0.9** | **0.80** | hsa-miR-505-3p | 1.3E-01 | -0.3 | 2.04 |
| **hsa-miR-15b-3p** | **6.5E-04** | **-0.9** | **1.33** | hsa-miR-30b-5p | 1.4E-01 | -0.2 | 3.22 |
| **hsa-miR-660-5p** | **8.4E-04** | **-0.5** | **2.69** | hsa-miR-30a-5p | 1.5E-01 | -0.2 | 1.68 |
| **hsa-miR-582-5p** | **1.2E-03** | **-1.0** | **1.96** | hsa-miR-181a-3p | 1.5E-01 | -0.2 | 3.09 |
| **hsa-miR-223-3p** | **1.2E-03** | **-0.9** | **4.07** | hsa-miR-22-3p | 1.5E-01 | -0.2 | 4.56 |
| **hsa-miR-340-5p** | **1.3E-03** | **-0.4** | **3.65** | hsa-miR-193a-5p | 1.7E-01 | -1.0 | 0.65 |
| **hsa-miR-340-3p** | **1.5E-03** | **-0.7** | **2.59** | hsa-miR-411-5p | 1.7E-01 | -0.6 | 0.83 |
| **hsa-miR-29a-3p** | **1.8E-03** | **-0.6** | **2.70** | hsa-miR-16-1-3p | 1.7E-01 | -0.3 | 0.69 |
| **hsa-miR-32-5p** | **2.0E-03** | **-0.9** | **1.31** | hsa-miR-100-5p | 1.7E-01 | -0.4 | 1.43 |
| **hsa-miR-5100** | **2.0E-03** | **-1.1** | **2.29** | hsa-miR-30e-5p | 1.8E-01 | -0.2 | 4.15 |
| **hsa-miR-1260b** | **2.5E-03** | **-0.7** | **3.40** | hsa-miR-99a-5p | 1.9E-01 | -0.3 | 0.80 |
| **hsa-miR-1260a** | **2.5E-03** | **-0.7** | **3.39** | hsa-miR-146a-5p | 1.9E-01 | -0.4 | 2.72 |
| **hsa-let-7f-1-3p** | **2.5E-03** | **-0.6** | **1.41** | hsa-miR-363-3p | 2.0E-01 | -0.2 | 2.61 |
| **hsa-miR-625-3p** | **2.5E-03** | **-0.6** | **1.41** | hsa-miR-378c | 2.0E-01 | -0.2 | 2.61 |
| **hsa-miR-532-5p** | **2.6E-03** | **-0.4** | **3.22** | hsa-let-7b-5p | 2.0E-01 | -0.4 | 3.16 |
| **hsa-miR-20a-3p** | **2.8E-03** | **-0.6** | **0.91** | hsa-miR-4802-3p | 2.0E-01 | -0.3 | 0.75 |
| **hsa-miR-142-3p** | **3.5E-03** | **-0.6** | **3.52** | hsa-miR-345-5p | 2.2E-01 | -0.1 | 3.30 |
| **hsa-miR-374b-5p** | **3.9E-03** | **-0.6** | **2.24** | hsa-miR-98-3p | 2.5E-01 | -0.4 | 1.70 |
| **hsa-miR-125b-5p** | **3.9E-03** | **-0.6** | **1.14** | hsa-miR-9-5p | 2.5E-01 | -0.2 | 1.69 |
| **hsa-miR-424-5p** | **4.0E-03** | **-0.7** | **1.86** | hsa-miR-574-5p | 2.5E-01 | -0.5 | 1.26 |
| **hsa-miR-338-3p** | **4.6E-03** | **-0.6** | **2.15** | hsa-miR-181c-3p | 2.5E-01 | -0.2 | 1.78 |
| **hsa-miR-16-2-3p** | **6.0E-03** | **-0.5** | **2.17** | hsa-miR-301a-3p | 2.5E-01 | -0.2 | 3.16 |
| **hsa-miR-378a-5p** | **8.5E-03** | **-0.4** | **1.60** | hsa-miR-132-5p | 2.6E-01 | -0.3 | 0.55 |
| **hsa-miR-26a-2-3p** | **9.8E-03** | **-0.8** | **1.19** | hsa-miR-106b-5p | 3.0E-01 | -0.2 | 2.78 |
| **hsa-miR-339-5p** | **1.1E-02** | **-0.4** | **2.05** | hsa-miR-378d | 3.1E-01 | -0.2 | 1.89 |
| **hsa-miR-574-3p** | **1.4E-02** | **-0.6** | **2.56** | hsa-miR-18a-5p | 3.1E-01 | -0.1 | 1.99 |
| **hsa-miR-28-5p** | **1.4E-02** | **-0.2** | **3.08** | hsa-miR-215-5p | 3.1E-01 | -0.3 | 0.96 |
| **hsa-miR-200a-3p** | **1.4E-02** | **-0.5** | **0.79** | hsa-miR-126-5p | 3.2E-01 | -0.3 | 2.50 |
| **hsa-miR-26b-5p** | **1.4E-02** | **-0.3** | **4.23** | hsa-miR-15a-5p | 3.2E-01 | -0.2 | 3.30 |
| **hsa-miR-142-5p** | **1.7E-02** | **-0.6** | **4.45** | hsa-miR-1304-3p | 3.2E-01 | -0.1 | 2.22 |
| **hsa-miR-185-5p** | **1.7E-02** | **-1.0** | **1.40** | hsa-miR-7705 | 3.3E-01 | -0.2 | 1.25 |
| **hsa-miR-30c-5p** | **2.1E-02** | **-0.3** | **3.96** | hsa-miR-5701 | 3.3E-01 | -0.4 | 1.56 |
| **hsa-miR-7977** | **2.4E-02** | **-0.8** | **2.64** | hsa-let-7b-3p | 3.3E-01 | -0.3 | 1.87 |
| **hsa-miR-101-3p** | **2.4E-02** | **-0.4** | **3.51** | hsa-miR-30d-5p | 3.3E-01 | -0.1 | 4.05 |
| **hsa-let-7a-3p** | **2.5E-02** | **-0.6** | **1.79** | hsa-miR-744-3p | 3.3E-01 | -0.2 | 1.05 |
| **hsa-miR-374b-3p** | **2.8E-02** | **-0.4** | **1.41** | hsa-miR-20a-5p | 3.5E-01 | -0.2 | 2.78 |
| **hsa-let-7f-2-3p** | **3.0E-02** | **-0.6** | **1.38** | hsa-miR-150-5p | 3.6E-01 | -0.3 | 3.43 |
| **hsa-miR-1249-3p** | **3.4E-02** | **-0.7** | **1.63** | hsa-miR-212-3p | 3.6E-01 | -0.2 | 0.63 |
| **hsa-miR-454-5p** | **3.8E-02** | **-0.4** | **2.01** | hsa-miR-141-3p | 3.7E-01 | -0.2 | 1.53 |
| **hsa-miR-582-3p** | **4.9E-02** | **-0.5** | **2.38** | hsa-miR-548w | 3.9E-01 | -0.2 | 1.01 |
| **hsa-let-7g-3p** | **4.9E-02** | **-0.4** | **0.73** | hsa-miR-365a-3p | 3.9E-01 | -0.2 | 1.45 |
|  |  |  |  | hsa-miR-365b-3p | 3.9E-01 | -0.2 | 1.45 |
|  |  |  |  | hsa-miR-331-3p | 3.9E-01 | -0.2 | 1.63 |
|  |  |  |  | hsa-miR-1306-5p | 3.9E-01 | -0.3 | 0.95 |
|  |  |  |  | hsa-miR-320c | 4.0E-01 | -0.2 | 1.05 |
|  |  |  |  | hsa-miR-532-3p | 4.0E-01 | -0.2 | 1.91 |
|  |  |  |  | hsa-miR-598-3p | 4.1E-01 | -0.2 | 0.93 |
|  |  |  |  | hsa-miR-409-3p | 4.1E-01 | -0.4 | 1.23 |
|  |  |  |  | hsa-miR-580-3p | 4.1E-01 | -0.2 | 0.84 |
|  |  |  |  | hsa-miR-486-3p | 4.1E-01 | -0.3 | 1.43 |
|  |  |  |  | hsa-miR-769-5p | 4.2E-01 | -0.1 | 2.94 |
|  |  |  |  | hsa-miR-548e-5p | 4.2E-01 | -0.1 | 1.58 |
|  |  |  |  | hsa-miR-181a-2-3p | 4.5E-01 | -0.1 | 2.69 |
|  |  |  |  | hsa-miR-200b-3p | 4.6E-01 | -0.1 | 1.89 |
|  |  |  |  | hsa-miR-542-5p | 4.7E-01 | -0.1 | 1.03 |
|  |  |  |  | hsa-miR-181b-3p | 4.8E-01 | -0.2 | 1.40 |
|  |  |  |  | hsa-miR-3613-3p | 4.9E-01 | -0.2 | 1.51 |
|  |  |  |  | hsa-miR-197-3p | 5.0E-01 | -0.3 | 2.67 |
|  |  |  |  | hsa-miR-93-3p | 5.2E-01 | -0.1 | 2.10 |
|  |  |  |  | hsa-miR-629-5p | 5.2E-01 | -0.2 | 1.24 |
|  |  |  |  | hsa-miR-361-3p | 5.2E-01 | -0.1 | 2.53 |
|  |  |  |  | hsa-miR-326 | 5.3E-01 | -0.1 | 1.82 |
|  |  |  |  | hsa-miR-641 | 5.3E-01 | -0.1 | 1.13 |
|  |  |  |  | hsa-miR-3143 | 5.5E-01 | -0.2 | 0.54 |
|  |  |  |  | hsa-miR-320a | 5.6E-01 | -0.2 | 2.71 |
|  |  |  |  | hsa-miR-27a-5p | 5.7E-01 | -0.2 | 2.54 |
|  |  |  |  | hsa-miR-424-3p | 5.7E-01 | -0.2 | 2.27 |
|  |  |  |  | hsa-miR-671-5p | 5.7E-01 | -0.1 | 1.10 |
|  |  |  |  | hsa-miR-7704 | 5.7E-01 | -0.2 | 0.91 |
|  |  |  |  | hsa-miR-6503-5p | 5.8E-01 | -0.1 | 2.01 |
|  |  |  |  | hsa-miR-2110 | 5.8E-01 | -0.2 | 0.76 |
|  |  |  |  | hsa-miR-502-5p | 5.9E-01 | -0.1 | 0.89 |
|  |  |  |  | hsa-miR-502-3p | 5.9E-01 | -0.1 | 1.92 |
|  |  |  |  | hsa-let-7d-3p | 6.1E-01 | -0.2 | 2.65 |
|  |  |  |  | hsa-miR-3157-3p | 6.2E-01 | -0.1 | 0.56 |
|  |  |  |  | hsa-let-7g-5p | 6.4E-01 | -0.1 | 3.84 |
|  |  |  |  | hsa-miR-766-3p | 6.5E-01 | -0.2 | 1.34 |
|  |  |  |  | hsa-miR-148b-5p | 6.6E-01 | -0.1 | 2.07 |
|  |  |  |  | hsa-let-7i-3p | 6.8E-01 | -0.1 | 1.08 |
|  |  |  |  | hsa-miR-27a-3p | 6.9E-01 | -0.1 | 3.58 |
|  |  |  |  | hsa-miR-146b-5p | 6.9E-01 | -0.1 | 3.23 |
|  |  |  |  | hsa-miR-193a-3p | 7.0E-01 | -0.1 | 1.35 |
|  |  |  |  | hsa-miR-320b | 7.2E-01 | -0.1 | 1.78 |
|  |  |  |  | hsa-miR-378f | 7.6E-01 | -0.1 | 1.18 |
|  |  |  |  | hsa-miR-3158-3p | 7.7E-01 | -0.1 | 0.89 |
|  |  |  |  | hsa-miR-30a-3p | 7.9E-01 | -0.1 | 1.53 |
|  |  |  |  | hsa-miR-30d-3p | 8.0E-01 | -0.1 | 2.12 |
|  |  |  |  | hsa-miR-4454 | 8.2E-01 | -0.1 | 2.18 |
|  |  |  |  | hsa-miR-125a-5p | 8.8E-01 | -0.1 | 1.98 |

*MiRs upregulated (IBD vs. HC)*

| **miR** | **FDR**  **<0.05** | **FC**  **log_2_** | **CPM**  **log_10_** | **miR** | **FDR**  **>0.05** | **FC**  **log_2_** | **CPM**  **log_10_** |
| --- | --- | --- | --- | --- | --- | --- | --- |
| **hsa-miR-7641** | **1.2E-09** | **1.7** | **1.33** | hsa-miR-32-3p | 5.1E-02 | 0.3 | 1.38 |
| **hsa-miR-92b-3p** | **7.2E-09** | **1.0** | **2.44** | hsa-miR-425-3p | 5.1E-02 | 0.3 | 2.37 |
| **hsa-miR-1285-3p** | **5.1E-07** | **0.9** | **1.92** | hsa-miR-155-5p | 5.1E-02 | 0.4 | 3.21 |
| **hsa-miR-27b-3p** | **5.1E-06** | **0.6** | **3.73** | hsa-miR-3605-5p | 5.2E-02 | 0.5 | 1.24 |
| **hsa-miR-21-3p** | **2.7E-05** | **0.8** | **3.78** | hsa-miR-505-5p | 5.4E-02 | 0.6 | 0.73 |
| **hsa-miR-3614-5p** | **2.7E-05** | **1.0** | **2.11** | hsa-miR-1287-5p | 5.5E-02 | 0.4 | 0.78 |
| **hsa-miR-196b-5p** | **4.1E-05** | **0.9** | **2.05** | hsa-miR-2115-3p | 5.5E-02 | 0.6 | 1.18 |
| **hsa-miR-30c-1-3p** | **6.7E-05** | **0.7** | **2.17** | hsa-miR-1303 | 7.4E-02 | 0.5 | 1.01 |
| **hsa-miR-34c-5p** | **1.3E-04** | **0.8** | **1.22** | hsa-miR-146b-3p | 7.5E-02 | 0.3 | 1.67 |
| **hsa-miR-671-3p** | **2.6E-04** | **0.5** | **2.65** | hsa-miR-148a-5p | 7.5E-02 | 0.5 | 1.75 |
| **hsa-miR-16-5p** | **3.1E-04** | **0.4** | **4.86** | hsa-miR-181a-5p | 7.8E-02 | 0.4 | 4.89 |
| **hsa-miR-191-5p** | **3.4E-04** | **0.4** | **4.92** | hsa-miR-550a-3p | 8.0E-02 | 0.3 | 1.23 |
| **hsa-miR-92a-1-5p** | **5.9E-04** | **1.0** | **1.47** | hsa-miR-1254 | 8.0E-02 | 0.5 | 0.68 |
| **hsa-miR-182-5p** | **6.6E-04** | **1.7** | **1.71** | hsa-miR-6818-5p | 9.2E-02 | 0.3 | 0.69 |
| **hsa-miR-185-3p** | **8.4E-04** | **0.5** | **1.68** | hsa-miR-6852-5p | 9.3E-02 | 0.4 | 1.05 |
| **hsa-miR-4677-3p** | **1.0E-03** | **0.4** | **1.70** | hsa-miR-484 | 9.5E-02 | 0.2 | 3.37 |
| **hsa-miR-500a-3p** | **1.1E-03** | **0.4** | **3.14** | hsa-miR-222-3p | 9.5E-02 | 0.3 | 3.65 |
| **hsa-miR-128-1-5p** | **1.5E-03** | **0.7** | **1.34** | hsa-miR-1273h-3p | 1.1E-01 | 0.3 | 2.06 |
| **hsa-miR-29c-5p** | **1.8E-03** | **0.5** | **1.48** | hsa-miR-29b-2-5p | 1.1E-01 | 0.3 | 0.88 |
| **hsa-miR-628-3p** | **1.8E-03** | **0.6** | **1.76** | hsa-miR-874-3p | 1.1E-01 | 0.4 | 0.90 |
| **hsa-miR-550a-5p** | **1.8E-03** | **0.6** | **1.15** | hsa-miR-145-5p | 1.1E-01 | 0.6 | 0.98 |
| **hsa-miR-550a-3-5p** | **1.8E-03** | **0.6** | **1.15** | hsa-miR-3615 | 1.1E-01 | 0.3 | 2.90 |
| **hsa-miR-941** | **2.3E-03** | **0.6** | **3.54** | hsa-miR-3605-3p | 1.2E-01 | 0.3 | 1.88 |
| **hsa-miR-6837-3p** | **2.4E-03** | **0.5** | **0.83** | hsa-miR-30b-3p | 1.2E-01 | 0.5 | 1.02 |
| **hsa-miR-421** | **2.6E-03** | **0.3** | **3.11** | hsa-miR-378g | 1.3E-01 | 0.4 | 1.07 |
| **hsa-let-7d-5p** | **2.6E-03** | **0.5** | **3.58** | hsa-miR-23a-3p | 1.3E-01 | 0.2 | 3.21 |
| **hsa-miR-186-5p** | **2.8E-03** | **0.2** | **4.01** | hsa-miR-7848-3p | 1.3E-01 | 0.4 | 0.86 |
| **hsa-miR-93-5p** | **3.4E-03** | **0.3** | **3.74** | hsa-miR-3074-5p | 1.3E-01 | 0.3 | 0.73 |
| **hsa-miR-2467-5p** | **3.6E-03** | **0.7** | **1.61** | hsa-miR-181b-5p | 1.3E-01 | 0.4 | 3.75 |
| **hsa-miR-130b-3p** | **3.8E-03** | **0.4** | **2.97** | hsa-miR-128-3p | 1.3E-01 | 0.2 | 3.36 |
| **hsa-miR-15b-5p** | **3.9E-03** | **0.3** | **2.95** | hsa-miR-4746-5p | 1.4E-01 | 0.3 | 1.26 |
| **hsa-miR-28-3p** | **3.9E-03** | **0.4** | **4.13** | hsa-miR-3909 | 1.5E-01 | 0.2 | 2.00 |
| **hsa-miR-371b-5p** | **3.9E-03** | **0.4** | **2.26** | hsa-miR-548l | 1.5E-01 | 0.3 | 0.85 |
| **hsa-miR-223-5p** | **3.9E-03** | **0.5** | **3.00** | hsa-miR-151a-3p | 1.6E-01 | 0.3 | 3.11 |
| **hsa-miR-130a-3p** | **3.9E-03** | **0.4** | **2.38** | hsa-miR-423-5p | 1.7E-01 | 0.5 | 3.77 |
| **hsa-miR-361-5p** | **6.2E-03** | **0.4** | **2.79** | hsa-miR-151b | 1.7E-01 | 0.3 | 2.95 |
| **hsa-miR-625-5p** | **1.4E-02** | **0.4** | **1.50** | hsa-miR-151a-5p | 1.7E-01 | 0.3 | 2.96 |
| **hsa-miR-21-5p** | **1.6E-02** | **0.3** | **5.03** | hsa-miR-330-3p | 1.7E-01 | 0.2 | 1.65 |
| **hsa-miR-627-5p** | **1.6E-02** | **0.4** | **1.36** | hsa-miR-501-3p | 1.7E-01 | 0.3 | 2.28 |
| **hsa-miR-4781-3p** | **1.8E-02** | **0.5** | **0.73** | hsa-miR-27b-5p | 1.7E-01 | 0.3 | 1.36 |
| **hsa-miR-191-3p** | **1.9E-02** | **0.3** | **2.14** | hsa-let-7a-5p | 1.8E-01 | 0.3 | 4.56 |
| **hsa-miR-942-5p** | **2.1E-02** | **0.4** | **2.11** | hsa-miR-1307-3p | 1.9E-01 | 0.3 | 2.71 |
| **hsa-miR-210-3p** | **3.0E-02** | **0.5** | **0.97** | hsa-miR-1538 | 1.9E-01 | 0.3 | 0.90 |
| **hsa-miR-330-5p** | **3.3E-02** | **0.3** | **1.73** | hsa-miR-548j-5p | 1.9E-01 | 0.4 | 1.04 |
| **hsa-let-7i-5p** | **3.3E-02** | **0.4** | **4.25** | hsa-miR-371b-3p | 1.9E-01 | 0.4 | 0.69 |
| **hsa-miR-6842-3p** | **3.4E-02** | **0.4** | **1.70** | hsa-miR-1296-5p | 2.0E-01 | 0.3 | 1.04 |
| **hsa-miR-188-5p** | **3.4E-02** | **0.5** | **1.67** | hsa-miR-33b-3p | 2.1E-01 | 0.3 | 0.86 |
| **hsa-miR-3174** | **3.4E-02** | **0.4** | **1.38** | hsa-miR-26a-5p | 2.2E-01 | 0.1 | 4.85 |
| **hsa-miR-1468-5p** | **3.9E-02** | **0.4** | **1.15** | hsa-miR-486-5p | 2.2E-01 | 0.5 | 2.83 |
|  |  |  |  | hsa-miR-143-3p | 2.3E-01 | 0.2 | 3.05 |
|  |  |  |  | hsa-miR-652-3p | 2.5E-01 | 0.3 | 2.50 |
|  |  |  |  | hsa-miR-877-5p | 2.5E-01 | 0.3 | 1.93 |
|  |  |  |  | hsa-miR-3176 | 2.7E-01 | 0.3 | 1.11 |
|  |  |  |  | hsa-miR-2277-5p | 2.8E-01 | 0.2 | 1.36 |
|  |  |  |  | hsa-miR-769-3p | 3.0E-01 | 0.3 | 0.86 |
|  |  |  |  | hsa-miR-98-5p | 3.0E-01 | 0.2 | 3.31 |
|  |  |  |  | hsa-miR-6502-5p | 3.0E-01 | 0.2 | 1.25 |
|  |  |  |  | hsa-miR-17-5p | 3.0E-01 | 0.1 | 2.93 |
|  |  |  |  | hsa-miR-940 | 3.2E-01 | 0.2 | 0.66 |
|  |  |  |  | hsa-miR-3690 | 3.2E-01 | 0.4 | 1.63 |
|  |  |  |  | hsa-miR-6511a-3p | 3.3E-01 | 0.3 | 0.75 |
|  |  |  |  | hsa-miR-362-5p | 3.5E-01 | 0.3 | 1.65 |
|  |  |  |  | hsa-miR-589-5p | 3.6E-01 | 0.1 | 2.34 |
|  |  |  |  | hsa-miR-339-3p | 3.9E-01 | 0.1 | 2.41 |
|  |  |  |  | hsa-miR-378i | 3.9E-01 | 0.1 | 2.07 |
|  |  |  |  | hsa-miR-30e-3p | 3.9E-01 | 0.2 | 3.37 |
|  |  |  |  | hsa-miR-148a-3p | 4.0E-01 | 0.2 | 3.92 |
|  |  |  |  | hsa-miR-7706 | 4.2E-01 | 0.2 | 2.08 |
|  |  |  |  | hsa-miR-26b-3p | 4.4E-01 | 0.2 | 2.68 |
|  |  |  |  | hsa-miR-324-5p | 4.8E-01 | 0.2 | 1.41 |
|  |  |  |  | hsa-miR-576-3p | 4.9E-01 | 0.1 | 1.36 |
|  |  |  |  | hsa-miR-423-3p | 4.9E-01 | 0.1 | 4.15 |
|  |  |  |  | hsa-miR-548at-5p | 4.9E-01 | 0.2 | 0.54 |
|  |  |  |  | hsa-miR-2116-3p | 4.9E-01 | 0.2 | 1.08 |
|  |  |  |  | hsa-miR-500b-5p | 5.0E-01 | 0.1 | 0.85 |
|  |  |  |  | hsa-miR-542-3p | 5.0E-01 | 0.2 | 1.61 |
|  |  |  |  | hsa-miR-744-5p | 5.2E-01 | 0.2 | 3.05 |
|  |  |  |  | hsa-miR-454-3p | 5.2E-01 | 0.1 | 2.79 |
|  |  |  |  | hsa-miR-664a-5p | 5.2E-01 | 0.2 | 0.88 |
|  |  |  |  | hsa-miR-3150b-3p | 5.2E-01 | 0.2 | 0.84 |
|  |  |  |  | hsa-miR-1301-3p | 5.3E-01 | 0.2 | 1.30 |
|  |  |  |  | hsa-miR-338-5p | 5.4E-01 | 0.2 | 1.60 |
|  |  |  |  | hsa-miR-25-5p | 5.4E-01 | 0.2 | 1.66 |
|  |  |  |  | hsa-miR-378a-3p | 5.5E-01 | 0.1 | 4.31 |
|  |  |  |  | hsa-miR-3613-5p | 5.5E-01 | 0.3 | 1.74 |
|  |  |  |  | hsa-miR-25-3p | 5.6E-01 | 0.1 | 4.06 |
|  |  |  |  | hsa-miR-584-5p | 5.6E-01 | 0.1 | 1.71 |
|  |  |  |  | hsa-miR-5010-3p | 5.7E-01 | 0.2 | 1.17 |
|  |  |  |  | hsa-miR-629-3p | 5.7E-01 | 0.2 | 0.72 |
|  |  |  |  | hsa-miR-1910-5p | 5.7E-01 | 0.2 | 0.82 |
|  |  |  |  | hsa-miR-422a | 5.9E-01 | 0.1 | 0.99 |
|  |  |  |  | hsa-miR-324-3p | 5.9E-01 | 0.1 | 1.30 |
|  |  |  |  | hsa-miR-551a | 5.9E-01 | 0.1 | 0.64 |
|  |  |  |  | hsa-miR-500a-5p | 6.0E-01 | 0.1 | 1.09 |
|  |  |  |  | hsa-miR-132-3p | 6.1E-01 | 0.1 | 1.68 |
|  |  |  |  | hsa-miR-103a-2-5p | 6.2E-01 | 0.1 | 0.64 |
|  |  |  |  | hsa-miR-4645-3p | 6.5E-01 | 0.1 | 1.17 |
|  |  |  |  | hsa-miR-874-5p | 6.6E-01 | 0.1 | 0.41 |
|  |  |  |  | hsa-miR-195-5p | 6.7E-01 | 0.1 | 0.53 |
|  |  |  |  | hsa-miR-501-5p | 6.8E-01 | 0.1 | 1.29 |
|  |  |  |  | hsa-miR-103a-3p | 6.8E-01 | 0.0 | 4.12 |
|  |  |  |  | hsa-miR-1307-5p | 6.8E-01 | 0.1 | 2.90 |
|  |  |  |  | hsa-miR-23b-3p | 7.0E-01 | 0.1 | 2.14 |
|  |  |  |  | hsa-miR-221-3p | 7.1E-01 | 0.1 | 3.20 |
|  |  |  |  | hsa-miR-181d-5p | 7.2E-01 | 0.1 | 2.05 |
|  |  |  |  | hsa-miR-140-3p | 7.3E-01 | 0.1 | 3.67 |
|  |  |  |  | hsa-miR-221-5p | 7.4E-01 | 0.1 | 1.99 |
|  |  |  |  | hsa-miR-548o-3p | 7.4E-01 | 0.1 | 1.60 |
|  |  |  |  | hsa-miR-6718-5p | 7.4E-01 | 0.1 | 1.19 |
|  |  |  |  | hsa-miR-10b-5p | 7.4E-01 | 0.1 | 0.89 |
|  |  |  |  | hsa-miR-200c-3p | 7.6E-01 | 0.1 | 1.22 |
|  |  |  |  | hsa-miR-548c-5p | 7.6E-01 | 0.0 | 1.25 |
|  |  |  |  | hsa-miR-548o-5p | 7.6E-01 | 0.0 | 1.25 |
|  |  |  |  | hsa-miR-548am-5p | 7.6E-01 | 0.0 | 1.25 |
|  |  |  |  | hsa-miR-548au-5p | 7.6E-01 | 0.0 | 1.25 |
|  |  |  |  | hsa-miR-190b | 7.7E-01 | 0.1 | 1.49 |
|  |  |  |  | hsa-miR-106b-3p | 7.7E-01 | 0.1 | 2.64 |
|  |  |  |  | hsa-miR-1284 | 8.0E-01 | 0.1 | 0.89 |
|  |  |  |  | hsa-miR-22-5p | 8.1E-01 | 0.1 | 1.24 |
|  |  |  |  | hsa-miR-548ay-5p | 8.3E-01 | 0.0 | 0.96 |
|  |  |  |  | hsa-miR-548k | 8.4E-01 | 0.0 | 2.34 |
|  |  |  |  | hsa-miR-7976 | 8.4E-01 | 0.1 | 0.57 |
|  |  |  |  | hsa-miR-301b-3p | 8.4E-01 | 0.0 | 2.43 |
|  |  |  |  | hsa-miR-6503-3p | 8.5E-01 | 0.0 | 2.43 |
|  |  |  |  | hsa-miR-194-5p | 8.7E-01 | 0.0 | 1.28 |
|  |  |  |  | hsa-miR-152-3p | 8.7E-01 | 0.0 | 1.84 |
|  |  |  |  | hsa-miR-192-5p | 8.8E-01 | 0.0 | 2.76 |
|  |  |  |  | hsa-miR-4511 | 8.8E-01 | 0.0 | 0.66 |
|  |  |  |  | hsa-miR-92a-3p | 8.8E-01 | 0.0 | 4.77 |
|  |  |  |  | hsa-miR-589-3p | 8.8E-01 | 0.0 | 1.73 |
|  |  |  |  | hsa-miR-425-5p | 8.8E-01 | 0.0 | 3.45 |
|  |  |  |  | hsa-let-7f-5p | 8.8E-01 | 0.0 | 5.01 |
|  |  |  |  | hsa-miR-3928-3p | 8.8E-01 | 0.0 | 0.68 |
|  |  |  |  | hsa-miR-10a-5p | 8.8E-01 | 0.0 | 2.53 |
|  |  |  |  | hsa-let-7c-5p | 8.8E-01 | 0.0 | 2.01 |
|  |  |  |  | hsa-miR-150-3p | 8.8E-01 | 0.1 | 0.92 |
|  |  |  |  | hsa-miR-664a-3p | 8.8E-01 | 0.0 | 1.63 |
|  |  |  |  | hsa-miR-628-5p | 8.8E-01 | 0.0 | 1.86 |
|  |  |  |  | hsa-miR-18b-5p | 9.0E-01 | 0.0 | 0.56 |
|  |  |  |  | hsa-miR-106a-5p | 9.0E-01 | 0.0 | 1.47 |
|  |  |  |  | hsa-miR-342-3p | 9.0E-01 | 0.0 | 2.87 |
|  |  |  |  | hsa-miR-503-5p | 9.1E-01 | 0.0 | 1.01 |
|  |  |  |  | hsa-miR-199a-3p | 9.3E-01 | 0.0 | 2.97 |
|  |  |  |  | hsa-miR-199b-3p | 9.3E-01 | 0.0 | 2.97 |
|  |  |  |  | hsa-miR-20b-5p | 9.4E-01 | 0.0 | 1.46 |
|  |  |  |  | hsa-miR-342-5p | 9.5E-01 | 0.0 | 1.67 |
|  |  |  |  | hsa-let-7e-5p | 9.5E-01 | 0.0 | 2.12 |
|  |  |  |  | hsa-miR-548d-5p | 9.5E-01 | 0.0 | 1.20 |
|  |  |  |  | hsa-miR-181c-5p | 9.5E-01 | 0.0 | 2.73 |
|  |  |  |  | hsa-miR-34a-5p | 9.5E-01 | 0.0 | 0.78 |
|  |  |  |  | hsa-miR-29a-5p | 9.7E-01 | 0.0 | 0.73 |
|  |  |  |  | hsa-miR-1306-3p | 9.7E-01 | 0.0 | 0.39 |
|  |  |  |  | hsa-miR-328-3p | 9.7E-01 | 0.0 | 2.06 |
|  |  |  |  | hsa-miR-3173-5p | 9.8E-01 | 0.0 | 1.23 |
|  |  |  |  | hsa-miR-331-5p | 9.9E-01 | 0.0 | 0.96 |
|  |  |  |  | hsa-miR-99b-5p | 1.0E+00 | 0.0 | 1.51 |
