# Supplementary material for: miR-374a-5p regulates inflammatory genes and monocyte function in patients with inflammatory bowel disease
Source: J Exp Med. 2022 Apr 1;219(5):e20211366. doi: 10.1084/jem.20211366 (PMC8980842; doi:10.1084/jem.20211366)
Supplement: Table S3 — shows a description of targets of miR-374a-5p. [file JEM_20211366_TableS3.docx]

**Table S3.** Description of targets of miR-374a-5p

| **miR-374**  **targets** | **Official**  **name** | **Class** | **Role in**  **IBD/inflammation** | **References** | **Genetic association**  **with IBD**  **(GWAS/references)** |
| --- | --- | --- | --- | --- | --- |
| IL6 | Interleukin 6 | Cytokine | Therapeutic target for IBD | Alloca et al. (2013) |  |
|  |  |  | Master regulator of intestinal  chronic inflammation | Waldner and Neurath (2014) |  |
| OSM | Oncostatin M | Cytokine | Therapeutic target for IBD | Thomas (2017) | Jostins et al. (2012) |
|  |  |  | Key cytokine driving inflammation in IBD | West et al. (2017) |  |
| CCL18 | C-C Motif Chemokine Ligand 18 | Cytokine | Recruitment of lymphocytes and dendritic cells | Schutyser et al. (2005) |  |
| IL1A | Interleukin 1 Alpha | Cytokine | Pro-inflammatory mediator of immune  response to intracellular pathogens | Malik and Kanneganti (2018) |  |
| PDE4B | Phosphodiesterase 4B | Immune  mediator | New therapeutic target for IBD | Spadaccini et al. (2017) |  |
|  |  |  | Metabolization of cAMP in inflammatory cells | Azam and Tripuraneni (2014) |  |
| PTGER4 | Prostaglandin E Receptor 4 | Immune  mediator | Therapeutic target for pain and inflammation | Shaw et al. (2015) | Libioulle et al. (2007) |
|  |  |  | Maturation and migration of immune cells | Kashmiry et al. (2018) |  |
| ZFP36 | ZFP36 Ring Finger Protein | Immune  mediator | Therapeutic target for inflammatory diseases | Patial and Blackshear (2016) |  |
|  |  |  | Master regulator of pro-inflammatory gene expression | Ross et al. (2017) |  |
| TNFAIP3 | TNF Alpha Induced Protein 3 | Immune  mediator | Regulator of NF-kappa B and TNF signaling | Vereecke et al. (2010) | Ellinghaus et al. (2016) |
| KLF6 | Kruppel Like Factor 6 | Transcription factor | Central regulator of pathogenic myeloid cells in IBD | Goodman et al. (2016) |  |
| NFKBIZ | NFKB Inhibitor Zeta | Transcription factor | Regulation of inflammatory genes and monocyte recruitment | Hildebrand et al. (2013) | Liu et al. (2015) |
|  |  |  | Critical regulator of Th17 development | Okamoto et al. (2010) |  |
| ZBTB10 | Zinc Finger and BTB Domain Containing 10 | Transcription  factor | Linked to asthma susceptibility  Regulates Sp1, a transcriptional activator of proinflammatory cytokines | Seumois et al. (2016) |  |
|  |  |  | Intestinal mucosa homeostasis | Salehi et al. (2012) | Barrett et al. (2008) |
| PRDM1 | PR/SET Domain 1 (Blimp1) | Transcription factor | Key regulator of T and B cell differentiation and function | Cretney et al. (2018) | Ellinghaus et al. (2016) |
|  |  |  |  |  | Anderson et al. (2011) |
|  |  |  |  |  | Franke et al. (2010) |
| OR2L13 | Olfactory Receptor Family 2 Subfamily L Member 13 | Unknown |  |  |  |
| KCNT2 | Potassium Sodium-Activated Channel Subfamily T Member 2 | Unknown |  |  |  |

References

Allocca, M., M. Jovani, G. Fiorino, S. Schreiber, and S. Danese. 2013. Anti-IL-6 treatment for inflammatory bowel diseases: next cytokine, next target. *Curr. Drug Targets*. 14:1508–1521. https://doi.org/10.2174/13894501113146660224

Anderson, C.A., G. Boucher, C.W. Lees, A. Franke, M. D’Amato, K.D. Taylor, J.C. Lee, P. Goyette, M. Imielinski, A. Latiano, et al. 2011. Meta-analysis identifies 29 additional ulcerative colitis risk loci, increasing the number of confirmed associations to 47. *Nat. Genet.* 43:246–252. https://doi.org/10.1038/ng.764

Azam, M.A., and N.S. Tripuraneni. 2014. Selective Phosphodiesterase 4B Inhibitors: A Review. *Sci. Pharm.* 82:453–481. https://doi.org/10.3797/scipharm.1404-08

Barrett, J.C., S. Hansoul, D.L. Nicolae, J.H. Cho, R.H. Duerr, J.D. Rioux, S.R. Brant, M.S. Silverberg, K.D. Taylor, M.M. Barmada, et al. 2008. Genome-wide association defines more than 30 distinct susceptibility loci for Crohn’s disease. *Nat. Genet.* 40:955–962. https://doi.org/10.1038/ng.175

Cretney, E., P.S. Leung, S. Trezise, D.M. Newman, L.C. Rankin, C.E. Teh, T.L. Putoczki, D.H. Gray, G.T. Belz, L.A. Mielke, et al. 2018. Characterization of Blimp-1 function in effector regulatory T cells. *J. Autoimmun.* 91:73–82. https://doi.org/10.1016/j.jaut.2018.04.003

Ellinghaus, D., L. Jostins, S.L. Spain, A. Cortes, J. Bethune, B. Han, Y.R. Park, S. Raychaudhuri, J.G. Pouget, M. Hübenthal, et al. 2016. Analysis of five chronic inflammatory diseases identifies 27 new associations and highlights disease-specific patterns at shared loc. *Nat. Genet.* 48:510–518. https://doi.org/10.1038/ng.3528

Franke, A., D.P.B. McGovern, J.C. Barrett, K. Wang, G.L. Radford-Smith, T. Ahmad, C.W. Lees, T. Balschun, J. Lee, R. Roberts, et al. 2010. Genome-wide meta-analysis increases to 71 the number of confirmed Crohn's disease susceptibility loci. *Nat. Genet.* 42:1118–1125. https://doi.org/10.1038/ng.717

Goodman, W.A., S. Omenetti, D. Date, L. Di Martino, C. De Salvo, G.-D. Kim, S. Chowdhry, G. Bamias, F. Cominelli, T.T. Pizarro, et al. 2016. KLF6 contributes to myeloid cell plasticity in the pathogenesis of intestinal inflammation. *Mucosal Immunol.* 9:1250–1262. https://doi.org/10.1038/mi.2016.1

Hildebrand, D.G., E. Alexander, S. Hörber, S. Lehle, K. Obermayer, N.-A. Münck, O. Rothfuss, J.-S. Frick, M. Morimatsu, I. Schmitz, et al. 2013. *J. Immunol.* 190:4812–4820. https://doi.org/10.4049/jimmunol.1300089

Jostins, L., S. Ripke, R.K. Weersma, R.H. Duerr, D.P. McGovern, K.Y. Hui, J.C. Lee, L.P. Schumm, Y. Sharma, C.A. Anderson, et al. 2012. Host-microbe interactions have shaped the genetic architecture of inflammatory bowel disease. *Nature*. 491:119–124. https://doi.org/10.1038/nature11582

Kashmiry, A., R. Tate, G. Rotondo, J. Davidson, D. Rotondo. 2018. The prostaglandin EP4 receptor is a master regulator of the expression of PGE 2 receptors following inflammatory activation in human monocytic cells. *Biochim. Biophys. Acta Mol. Cell Biol. Lipids.* 1863:1297–1304. https://doi.org/10.1016/j.bbalip.2018.07.003

Libioulle, C., E. Louis, S. Hansoul, C. Sandor, F. Farnir, D. Franchimont, S. Vermeire, O. Dewit, M. de Vos, A. Dixon, et al. 2007. Novel Crohn disease locus identified by genome-wide association maps to a gene desert on 5p13.1 and modulates expression of PTGER4. *PLoS Genet.* 3:e58. https://doi.org/10.1371/journal.pgen.0030058

Liu, J.Z., S. van Sommeren, H. Huang, S.C. Ng, R. Alberts, A. Takahashi, S. Ripke, J.C. Lee, L. Jostins, T. Shah, et al. 2015. Association analyses identify 38 susceptibility loci for inflammatory bowel disease and highlight shared genetic risk across populations. *Nat. Genet.* 47:979–986. https://doi.org/10.1038/ng.3359

Malik, A., and T.-D. Kanneganti. 2018. Function and regulation of IL-1α in inflammatory diseases and cancer. *Immunol. Rev.* 281:124–137. https://doi.org/10.1111/imr.12615

Okamoto, K., Y. Iwai, M. Oh-Hora, M. Yamamoto, T. Morio, K. Aoki, K. Ohya, A.M. Jetten, S. Akira, T. Muta, et al. 2010. IkappaBzeta regulates T(H)17 development by cooperating with ROR nuclear receptors. *Nature*. 464:1381–1385. https://doi.org/10.1038/nature08922

Patial, S., and P.J. Blackshear. 2016. Tristetraprolin as a Therapeutic Target in Inflammatory Disease. *Trends Pharmacol. Sci.* 37:811–821. https://doi.org/10.1016/j.tips.2016.07.002

Ross, E.A., A.J. Naylor, J.D. O’Neil, T. Crowley, M.L. Ridley, J. Crowe, T. Smallie, T.J. Tang, J.D. Turner, L.V. Norling, et al. 2017. Treatment of inflammatory arthritis via targeting of tristetraprolin, a master regulator of pro-inflammatory gene expression. *Ann. Rheum. Dis.* 76:612–619. https://doi.org/10.1136/annrheumdis-2016-209424

Salehi, S., R. Bankoti, L. Benevides, J. Willen, M. Couse, J.S. Silva, D. Dhall, E. Meffre, S. Targan, and G.A. Martins. 2012. B lymphocyte-induced maturation protein-1 contributes to intestinal mucosa homeostasis by limiting the number of IL-17-producing CD4+ T cells. *J. Immunol.* 189:5682–5693. https://doi.org/10.4049/jimmunol.1201966

Schutyser, E., A. Richmond, and J. Van Damme. 2005. Involvement of CC chemokine ligand 18 (CCL18) in normal and pathological processes. *J. Leukoc. Biol.* 78:14–26. https://doi.org/10.1189/jlb.1204712

Seumois, G., J. Zapardiel-Gonzalo, B. White, D. Singh, V. Schulten, M. Dillon, D. Hinz, D.H. Broide, A. Sette, B. Peters, et al. 2016. Transcriptional Profiling of Th2 Cells Identifies Pathogenic Features Associated with Asthma. *J. Immunol.* 197:655–664. https://doi.org/10.4049/jimmunol.1600397

Shaw, K.K., L.C. Rausch-Derra, and L. Rhodes. 2015. Grapiprant: an EP4 prostaglandin receptor antagonist and novel therapy for pain and inflammation. *Vet. Med. Sci.* 2:3–9. https://doi.org/10.1002/vms3.13

Spadaccini, M., S. D’Alessio, L. Peyrin-Biroulet, and S. Danese. 2017. PDE4 Inhibition and Inflammatory Bowel Disease: A Novel Therapeutic Avenue. *Int. J. Mol. Sci.* 18:1276. https://doi.org/10.3390/ijms18061276

Thomas, H. 2017. IBD: Oncostatin M promotes inflammation in IBD. *Nat. Rev. Gastroenterol. Hepatol.* 14:261. https://doi.org/10.1038/nrgastro.2017.47

Vereecke, L., M. Sze, C. Mc Guire, B. Rogiers, Y. Chu, M. Schmidt-Supprian, M. Pasparakis, R. Beyaert, and G. van Loo. 2010. Enterocyte-specific A20 deficiency sensitizes to tumor necrosis factor-induced toxicity and experimental colitis. *J. Exp. Med.* 207:1513–1523. https://doi.org/10.1084/jem.20092474

Waldner, M.J., and M.F. Neurath. 2014. Master regulator of intestinal disease: IL-6 in chronic inflammation and cancer development. *Semin. Immunol.* 26:75–79. https://doi.org/10.1016/j.smim.2013.12.003

West, N.R., A.N. Hegazy, B.M.J. Owens, S.J. Bullers, B. Linggi, S. Buonocore, M. Coccia, D. Görtz, S. This, K. Stockenhuber, J. Pott, et al. 2017. Oncostatin M drives intestinal inflammation and predicts response to tumor necrosis factor–neutralizing therapy in patients with inflammatory bowel disease. *Nat. Med.* 23:579–589. https://doi.org/10.1038/nm.4307
